# Interface-Driven Fatigue Failure: Quantitative Assessment of Laser-Remanufactured Inconel 625 Coatings Under Dry Rolling/Sliding Contact

**DOI:** 10.3390/ma19194195

**Published:** 2026-09-30

**Authors:** Chen Zhou, Xin Wang, Wei Zhou, Linlin Liu, Maolin Luo, Fei Liu

**Affiliations:** 1 School of Integrated Circuits, Chongqing University of Posts and Telecommunications, Chongwen Road 2, Nan’an District, Chongqing 400065, China; 2 Hubei Institute of Water Resources Survey and Design Co., Ltd., Wuhan 430070, China

**Keywords:** laser remanufacturing, heterogeneous interface, rolling contact fatigue, interfacial critical damage

## Abstract

**Highlights:**

1. The relationship between material matching factors and the critical damage state of interfaces has been clarified.
2. The nominal interfacial shear strength and critical damage models have been established.
3. A quantitative basis for assessing the dry rolling/sliding contact performance of remanufactured coatings has been developed.

**What are the main findings?**

- A model for evaluating the rolling contact fatigue (dry rolling/sliding contact) performance of laser remanufactured In625 coatings based on interface damage has been established. This model reveals the differentiated patterns of the critical damage state (***D_C_***) at the interface, which correspond to various service-induced damage levels in the substrates.
- Based on the test results of five typical matrix dry rolling/sliding contacts, it has been verified that the fatigue life of the coating is jointly determined by the coupling effect of surface peak contact stress and the cumulative effect of interfacial shear stress. Furthermore, it is clarified that the interaction of the interfacial stress field is the core element regulating the fatigue service life of remanufactured components.

**What are the implications of the main findings?**

- This evaluation system provides a quantifiable analysis tool for the process optimization of laser remanufacturing of In625 coatings, which can support the selection of remanufacturing processes and life prediction for damaged components of high-end equipment.
- Subsequently, multi-physics coupling can be introduced to further expand the model’s applicability in extreme service environments, thereby providing more comprehensive theoretical support for the engineering application of laser remanufacturing.

**Abstract:**

Remanufacturing damaged components with high-performance materials often produces heterogeneous coating–substrate interfaces, which can substantially influence the rolling contact fatigue performance of repaired components. In this study, the fatigue behavior of laser-remanufactured Inconel 625 (In625) coatings was systematically investigated using different substrate materials. Particular attention was paid to the effects of substrate selection and interfacial heterogeneity on crack initiation, interfacial damage accumulation, and cyclic contact failure under dry rolling-contact conditions. Two quantitative models were developed to evaluate the nominal strength and critical damage of the coating–substrate systems, respectively. The proposed models were validated against available interfacial failure and experimental data, and the relative fatigue performance of five In625/substrate systems was comparatively assessed. Rolling-contact-fatigue tests were subsequently conducted using different substrate materials, followed by detailed fractographic characterization of the coating surfaces and coating–substrate interfaces. The experimental results showed good agreement with the model predictions, confirming the effectiveness of the proposed evaluation framework. The results indicate that the fatigue failure behavior of laser-remanufactured In625 coatings is governed by the competition between fatigue damage accumulation induced by the maximum surface contact stress and interfacial damage accumulation associated with the maximum interfacial shear stress. Furthermore, the relationship between material-matching factors and interfacial critical damage was clarified. Based on the established interfacial fatigue-damage evolution model, the effects of material-matching factors on interfacial fatigue life were quantitatively determined. The findings demonstrate that substrate selection and heterogeneous interface properties play decisive roles in controlling crack initiation and cyclic contact failure in laser-remanufactured In625 coatings under the investigated dry-contact conditions.

## 1. Introduction

Rolling-bearing raceways operate under repeated, highly concentrated and frequently combined rolling–sliding contact. During service, raceway damage may arise from wear, plastic deformation, surface roughening, pitting, spalling, and crack propagation. Once the damaged region exceeds the allowable repair limit, replacement of the entire component is often costly and resource-intensive. Laser-based remanufacturing has therefore attracted increasing attention as a localized repair strategy because it can selectively add material to damaged regions, reduce the heat input compared with conventional fusion processes, and enable the restoration of complex functional surfaces. The issue of metallurgical incompatibility impedes the formation of reliable bonding joints across more than 5500 commercial alloys [1], compelling the field of surface repair to rely on a restricted array of broad-spectrum materials, encompassing iron-based [2], nickel-based [3], and cobalt-based [4] options. Nevertheless, the performance of a laser-remanufactured raceway is determined not only by the properties of the deposited material, but also by the thermal history, microstructural heterogeneity, mechanical mismatch, and failure behavior of the coating/substrate interface.

Previous research on laser-based and hybrid manufacturing has mainly developed along three related but distinct directions. The first direction concerns the development of laser-based or hybrid manufacturing strategies for the fabrication and repair of bearing raceways and other highly loaded functional surfaces [5,6]. Laser wire cladding of martensitic chromium–silicon steel on an S235 substrate has been investigated in combination with subsequent rolling-contact testing [7]. This type of material combination represents a typical repaired raceway architecture in which a hard and wear-resistant functional layer is deposited onto a relatively low-alloy structural substrate. Other studies have examined laser repair of nickel-based superalloys and the fatigue response of the repaired region [8]. Researchers have employed the three-dimensional digital image correlation technique to investigate the three-point bending fatigue behavior of laser-repaired high-temperature alloy materials [9]. Their results demonstrate the importance of process-induced microstructural and mechanical changes in determining the fatigue response of laser-repaired regions. Laser cladding has also been applied to the remanufacturing of mining components, including composite coatings for conical picks [10]. These studies confirm the applicability of laser-based deposition to the repair or functionalization of heavily loaded components, but they generally focus on deposition quality, wear resistance, or the fatigue behavior of the deposited material itself. The specific relationship between the laser-deposited coating/substrate interface and rolling-contact-fatigue failure remains insufficiently established.

The second research direction concerns the characterization of the resulting metallurgical interface. A laser-remanufactured coating/substrate system should not be regarded as a homogeneous material because the coating and substrate generally differ in elastic modulus, yield strength, hardness, thermal conductivity, coefficient of thermal expansion, and plastic-deformation capacity [11]. These differences affect heat transfer during deposition, solidification and dilution, residual-stress generation, and stress redistribution under subsequent mechanical loading. The thermal history is particularly important because rapid heating and cooling can produce thermal gradients and constrained contraction near the fusion boundary [12]. If the coating and substrate have different coefficients of thermal expansion, the resulting thermal mismatch can generate tensile or compressive residual stresses at or near the interface [13]. Under cyclic contact loading, these residual stresses interact with the normal pressure and tangential traction generated by the rolling contact.

Interface properties therefore play a decisive role in controlling crack initiation and cyclic contact failure in laser-remanufactured In625 coatings. A mechanically strong coating may nevertheless exhibit poor rolling-contact performance if the interface has insufficient shear or normal strength. Conversely, a substrate with lower nominal strength may provide better fatigue resistance when its thermal and mechanical properties result in a more favorable interfacial stress state. The local interface behavior is also affected by dilution, epitaxial growth, elemental redistribution, interfacial defects, pores, lack-of-fusion regions, and the transition from the coating microstructure to the substrate microstructure [14,15]. These features may act as local stress concentrators and promote crack initiation either within the coating, along the interface, or in the substrate.

The third research direction focuses on the experimental and statistical evaluation of rolling-contact performance. In conventional homogeneous bearing materials, rolling-contact-fatigue behavior is commonly related to the maximum orthogonal shear stress, contact pressure, material hardness, surface condition, and statistically distributed material defects [16]. Surface roughness is an important factor because asperity interactions can modify the real contact area and generate local stress concentrations [17]. Researchers have explored the rolling contact fatigue failure mechanism of bearing steel under varying surface roughness conditions and heavy-load operating scenarios [18]. Their results emphasize that surface topography can influence the location and mechanism of crack initiation and should not be treated as an independent surface-quality parameter. In laser-remanufactured coatings, this issue is further complicated by the finite coating thickness, the mechanically dissimilar substrate, and the presence of an interface that may provide a preferential crack path.

In addition to experimental testing, statistical and numerical approaches have been developed to predict rolling-contact-fatigue damage and service life. It proposed a prediction method for rolling-contact-fatigue damage in pearlitic rail materials by combining shakedown limits, rough-set theory, and a cloud model [19]. Such approaches are valuable for describing the variability of fatigue damage and for incorporating multiple influencing parameters. However, models developed for homogeneous rail or bearing materials cannot directly represent the mixed-mode fracture behavior of a finite-thickness coating bonded to a dissimilar substrate. Similarly, numerical modelling of laser-clad surfaces can reproduce the deposited geometry and surface morphology, but it does not necessarily provide a failure criterion for the coating/substrate interface [20]. Several researchers have conducted studies on the modeling and simulation of the ground surface morphology of laser cladding in-situ reinforced nickel-based composite coatings [21,22,23]. This type of work is relevant to the description of the manufactured surface and its subsequent finishing condition, but surface morphology prediction alone cannot determine whether cyclic damage will initiate in the coating, at the interface, or in the substrate.

Existing rolling-contact-fatigue criteria therefore have several limitations when applied to laser-remanufactured coatings [24,25]. First, criteria based on the maximum orthogonal shear stress are generally formulated for homogeneous materials and do not explicitly distinguish coating failure from interfacial failure or substrate failure. Second, conventional contact-fatigue models usually do not incorporate the finite thickness of the coating or the discontinuity of normal and shear deformation across a mechanically heterogeneous interface. Third, residual stresses generated during laser deposition are often considered separately from the cyclic stress field produced by rolling contact, even though their interaction may strongly affect crack initiation. Fourth, static interface-strength measurements, such as tensile, shear, or bending tests, do not directly describe the cyclic mixed-mode loading condition experienced by the interface during rolling contact. Finally, many existing models predict fatigue damage or service life without explicitly introducing an interfacial critical-damage parameter that can be related to coating/substrate compatibility.

The present study addresses these limitations by establishing an interface-oriented framework for evaluating the rolling-contact performance of laser-remanufactured In625 coatings on different substrates. The framework contains two quantitative components. The first is a nominal interfacial-strength model that considers the mechanical and thermal incompatibility between the coating and substrate, including the shear yield strength, thermal-expansion mismatch, and strength-related parameters of the two materials. The second is a critical-damage model that describes the mixed-mode interfacial crack-driving force using the normal and shear components of the interfacial stress field and their corresponding stress-intensity factors. These two components are subsequently coupled with the cyclic contact stress field to determine the equivalent interfacial shear stress and the critical damage state. In contrast to models based solely on the bulk coating properties or the maximum subsurface shear stress, the proposed framework explicitly treats the coating/substrate boundary as a mechanically meaningful region. It connects the following sequence of processes: laser-induced thermal history→coating/substrate incompatibility→interfacial strength→mixed-mode crack driving force→critical damage→rolling-contact-fatigue response. The framework is evaluated using the available interfacial-failure and rolling-contact-fatigue data for In625 coatings deposited on different engineering substrates. The experimental matrix includes multiple steel, stainless-steel, nickel-based-alloy, and cast-iron substrates, thereby allowing the influence of substrate type on interfacial strength, residual stress, crack initiation, and rolling-contact performance to be compared. Because the bending and rolling-contact tests were not conducted on exactly the same set of coating/substrate combinations, the degree of experimental validation differs among the systems. Accordingly, the present study is intended to establish a comparative and physically interpretable framework for assessing interface-controlled rolling-contact damage, rather than a universal bearing-life model applicable to all coating thicknesses, lubrication conditions, surface finishes, and service loads. This work enhances the understanding of microstructural interactions at varied interfaces and provides valuable tools for improving material selection in laser remanufacturing processes.

## 2. Materials and Methods

### 2.1. Modeling of Nominal Interfacial Shear Strength

Layered structures of finite thickness, known as heterogeneous interfaces, are created through rapid non-equilibrium metallurgical processes. The measurement of intrinsic interface strength, which refers to the fundamental mechanical properties of the interfacial phase material, is particularly difficult due to physicochemical reactions and effects at the nanoscale. As a result, the nominal interface strength is determined by the stress needed to cause failure at the interface per unit area, serving as a useful measure for engineering purposes. In remanufactured coatings, where the thickness of the coating is much smaller than that of the substrate, traditional methods for evaluating interface strength fall short because of experimental constraints and the closure of cracks induced by compressive stress. Under dry rolling/sliding contact conditions, shear stress plays a more critical role in crack propagation at interfaces compared to normal stress.

Interfacial regions consist of diluted mixtures of repair materials and substrates, allowing for the establishment of a connection between core physical parameters at the interface and the nominal shear strength, which helps quantify the effects of material compatibility. Coatings and substrates behave like laminated plate structures, where differences in properties lead to strain mismatches, resulting in interface failure during plastic deformation. The thermo-physical parameters play a crucial role in determining interfacial stress fields; specifically, the mismatch in thermal expansion coefficients (Δα) influences the orientation of residual stresses near the interface, which in turn affects critical failure stress states. Heterogeneous interfaces, formed from metallurgically bonded ductile materials, follow the principles of welding compatibility and dimensional similarity theory. As a result, a model for nominal interface strength includes essential parameters such as shear yield strength (τ_is_), thermal expansion coefficient (α_i_), and compressive strength ratio (σ_ib_/σ_is_), as outlined in Equation (1).

(1) $$\tilde{\tau_{C}}=a_{1}\tau_{1s}+a_{2}\tau_{2s}+a_{3}\left(\alpha_{1}-\alpha_{2}\right)T_{1}\frac{\sigma_{1b}}{\sigma_{1s}}+a_{4}\left(\alpha_{1}-\alpha_{2}\right)T_{2}\frac{\sigma_{2b}}{\sigma_{2s}}$$

In this context, τ_is_ = 0.5σ_is_. Subscripts 1 and 2 represent the Inconel 625 (In625) coating and its underlying substrate, respectively. The nominal interface strength, which is defined as the critical failure strength when subjected to concentrated shear forces, quantitatively assesses the ability to resist interfacial shear. This metric is essential for analyzing the static mechanical behavior of heterogeneous interfaces composed of different material pairings.

### 2.2. Critical Damage of Heterogeneous Interface Under Dry Rolling/Sliding Contact

**Figure 1:**
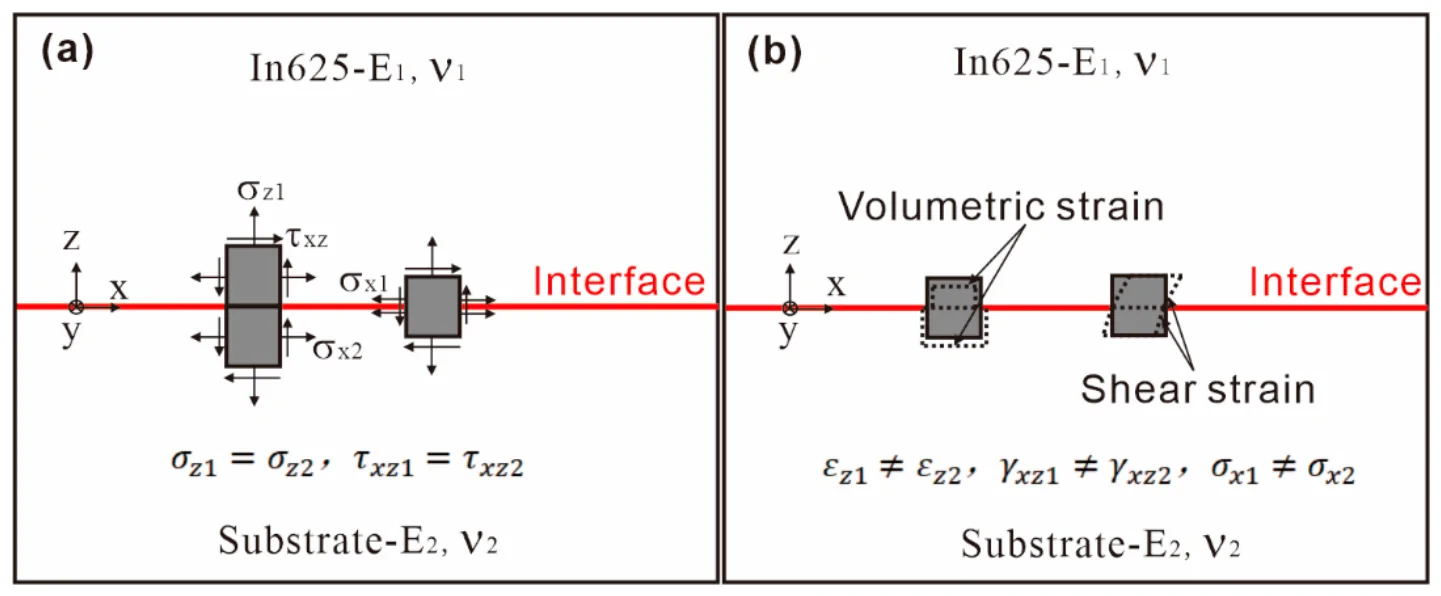
Stress (**a**) and strain (**b**) on both sides of heterogeneous interface.

Establishing a model for evaluation was crucial to measure how a heterogeneous interface affects the performance of rolling contact fatigue. In Figure 1, the mechanical model represents the interface as a boundary with no thickness, effectively illustrating the critical property discontinuity between different materials. This theoretical interface reflects the strength attributes of the interfacial phase, which are primarily determined by the mechanical characteristics of both the In625 coating and the substrate materials. From the viewpoint of continuum mechanics, the interface ensures continuity in surface tractions and displacements (σ_z1_ = σ_z2_, τ_xz1_ = τ_xz2_, u_1_ = u_2_, υ_1_ = υ_2_), while showing discontinuities in three significant parameters: the normal strain that is perpendicular to the interface (ε_z1_ ≠ ε_z2_), the shear strain (γ_xz1_ ≠ γ_xz2_), and the normal stress that runs parallel to the interface (σ_x1_ ≠ σ_x2_). These discontinuities stem directly from the differences in physical properties at the material boundary.

The failure of the interface was caused not just by basic peeling or shear cracking, but rather by the combined effects of both mechanisms. This interaction resulted in a variation in the stress intensity factor at the tip of the interfacial crack (Equation (2)) when subjected to far-field tensile (σ_0_) and shear stresses (τ_0_), differing from that observed in homogeneous materials [17].

(2) $$K_{1}+iK_{2}=\left(\sigma_{0}+i\tau_{0}\right)\sqrt{\pi a}\left(1+2\varepsilon i\right){\left(\frac{\rho }{2}\right)}^{i\varepsilon }$$

***K*_1_** and ***K*_2_** represented the intensity factors for peel and shear stress, differing from the stress fields associated with modes I and II ($K_{2C}\neq \sqrt{3}K_{1C}/2$). Here, the symbol a indicated the crack’s half-length, while ***ρ*** denoted the correlation coefficient and ***ε*** referred to the bi-material constant. The theoretical values for ***K*_1_** and ***K*_2_** could be derived from Equation (3) when the imaginary component equaled zero and ***ρ*** was set at 0.28.

(3) $$K_{1}=\sqrt{\pi a}\left[\sigma_{0}-2\varepsilon \tau_{0}\right],K_{2}=\sqrt{\pi a}\left[\tau_{0}+2\varepsilon \sigma_{0}\right]$$

By considering the physical characteristics of the interface phase as a comparable approach, the interfacial strain energy was defined in relation to the strain energy of the In625 material and its substrate, as illustrated in Equations (4) and (5) [8].

(4) $$\{\begin{array}{c} W_{N}=\frac{S_{N}}{32\pi r\;\cosh^{2}\left(\varepsilon \pi \right)}\left[\frac{\eta_{1}}{\mu_{1}}e^{-2\pi \varepsilon }+\frac{\eta_{2}}{\mu_{2}}e^{2\pi \varepsilon }\right]\\ W_{S}=\frac{S_{S}}{32\pi r\;\cosh^{2}\left(\varepsilon \pi \right)}\left(\frac{1}{\mu_{1}}+\frac{1}{\mu_{2}}\right)\left(e^{2\pi \varepsilon }+e^{-2\pi \varepsilon }+2\right) \end{array}$$

(5) $$\{\begin{array}{c} S_{N}=\left(K_{1}^{2}+K_{2}^{2}\right)\cos^{2}\left(\Upsilon +\varepsilon \ln\frac{r}{\rho a}\right)\\ S_{S}=\left(K_{1}^{2}+K_{2}^{2}\right)\left[1+C_{0}\cos^{2}\left(\Upsilon +\varepsilon \ln\frac{r}{\rho a}\right)\right]\\ C_{0}=\frac{\left[\frac{2\left(\kappa_{1}-1\right)-\eta_{1}}{\mu_{1}}e^{-2\pi \varepsilon }+\frac{2\left(\kappa_{2}-1\right)-\eta_{2}}{\mu_{2}}e^{2\pi \varepsilon }-4\left(\frac{1}{\mu_{1}}+\frac{1}{\mu_{2}}\right)\right]}{\left[\left(\frac{1}{\mu_{1}}+\frac{1}{\mu_{2}}\right)\left(e^{2\pi \varepsilon }+e^{-2\pi \varepsilon }+2\right)\right]} \end{array}$$

In this context, the subscripts N and S denote the energy associated with volume change strain and distortion, respectively. Under plane stress, Υ = arctan (K_2_/K_1_), η_j_ = 8(1 − 2υ_j_)/3(1 + υ_j_). Assuming that the strain energy for both types has attained a critical threshold, the criterion for crack propagation along the interface can be expressed in Equation (6).

(6) $$\frac{\left(K_{1}^{2}+K_{2}^{2}\right)\cos^{2}\left(\Upsilon +\varepsilon \ln\frac{r}{\rho a}\right)}{K_{1C}^{2}}+\frac{\left(K_{1}^{2}+K_{2}^{2}\right)\left[1+C_{0}\cos^{2}\left(\Upsilon +\varepsilon \ln\frac{r}{\rho a}\right)\right]}{K_{2C}^{2}}=1$$

In the case of interface fractures, cos^2^(εln(r/ρa)) ≈ 1, which are confined to the primary area of a singular field, the equation can be reduced to Equation (7).

(7) $${\left(\frac{K_{1}}{K_{1C}}\right)}^{2}+{\left(\frac{K_{2}}{K_{2C}}\right)}^{2}+{C\left(\frac{K_{1}}{K_{2C}}\right)}^{2}=1$$

(8) $$C=1+C_{0}=\;\frac{\frac{2\left(\kappa_{1}-1\right)-\eta_{1}}{\mu_{1}}e^{-2\pi \varepsilon }+\frac{2\left(\kappa_{2}-1\right)-\eta_{2}}{\mu_{2}}e^{2\pi \varepsilon }+\left(\frac{1}{\mu_{1}}+\frac{1}{\mu_{2}}\right)\left(e^{2\pi \varepsilon }+e^{-2\pi \varepsilon }-2\right)}{\left(\frac{1}{\mu_{1}}+\frac{1}{\mu_{2}}\right)\left(e^{2\pi \varepsilon }+e^{-2\pi \varepsilon }-2\right)}$$

The shear elastic modulus of the materials on either side is denoted μ_i_ = E_i_/[2(1 + ν_i_)], along with the kappa parameter (κ_i_). It is evident that the compatibility of materials influences, firstly, the critical failure values ***K*_1*C*_** and ***K*_2*C*_** of the interfacial phase materials, and secondly, it defines the contribution coefficient ***C*** that represents the normal stress related to interfacial shear failure.

The combined effects of mixed-mode loading significantly change the critical failure criteria at the interface. Although the maximum orthogonal shear stress criterion is still relevant for analyzing contact fatigue in uniform materials, repair coatings display unique failure behaviors. In these heterogeneous systems, maximum shear stress tends to accumulate in subsurface areas, which greatly enhances the likelihood of fatigue crack formation at the interfaces. In dry rolling/sliding contact scenarios, the presence of compressive normal stress helps keep interfacial cracks closed, making the mode I stress intensity factor (***K*_1_**) insignificant for crack growth. As a result, the initiation of interface cracks is primarily influenced by the mode II stress intensity factor (***K*_2_**), which aligns with conditions of maximum shear stress. The process of fatigue failure unfolds in three distinct stages: (1) initial crack growth occurs solely along the interface, (2) a shift to propagation on the coating side happens once critical static strength is achieved, and (3) the final stage involves delamination of the coating.

Significantly, the critical damage at the interface shifts from being governed by contact stress to being influenced by slip cracking. The primary fatigue parameter is defined by the modified range of shear stress intensity factor (∆***K*_2_**), which is obtained from the mixed-mode stress intensity factor in response to surface resultant forces. The criterion for crack propagation at the interface is outlined in Equation (9).

(9) $${\left(\frac{K_{2a}}{K_{2C}}\right)}^{2}+{C\left(\frac{K_{1a}}{K_{2C}}\right)}^{2}=1$$

(10) $$K_{1a}=\sqrt{\pi a}\left[\sigma_{a}-2\varepsilon \tau_{a}\right],K_{2a}=\sqrt{\pi a}\left[\tau_{a}+2\varepsilon \sigma_{a}\right]$$

In Equation (10), the cyclic normal (σ_a_) and shear stress (τ_a_) amplitudes are denoted. The characteristic length was defined as ***a*** = 1 μm. The parameters ***K*_1*a*_** and ***K*_2*a*_** represent the amplitudes of the interfacial peel and shear stress intensity factors, respectively. ***K*_2*C*_** indicates the shear resistance at the interface crack, which can be assessed based on the critical normal and shear stresses at the crack tip during three-point bending tests. The process of interfacial fatigue failure reveals a fundamental duality between the complexity of the microstructure and the overall strength characteristics. From the viewpoint of continuum damage mechanics, the interface experiences a gradual decline in strength linked to the accumulation of damage, leading to fatigue failure when the damage state reaches the critical threshold of bearing capacity, ***D_C_***. Key mechanistic insights include the following:

- The interfacial fatigue lifespan is primarily influenced by multiaxial fatigue characteristics, with the ultimate failure appearing as a slow, static fracture.
- The failure mechanism converges with homogeneous material behavior when static failure contributions are negligible.
- The interfacial fatigue response can be effectively characterized using equivalent fatigue stress formalism.

The essential failure metrics are defined as follows: the critical damage condition ***D_C_*** as presented in Equation (11), and the interfacial equivalent shear stress at the point of failure **[τ_C_]** as outlined in Equation (12).

(11) $$D_{C}=1-\sqrt{1/\left[{\left(\frac{K_{2a}}{K_{2C}}\right)}^{2}+{C\left(\frac{K_{1a}}{K_{2C}}\right)}^{2}\right]}$$

(12) $$\left[\tau_{C}\right]=\frac{\left[\tau \right]}{1-D_{C}}$$

Variations in substrate compatibility resulted in alterations to the physical characteristics of the interface phase, subsequently affecting the critical damage state ***D_C_***. When the interfacial stress intensity factor remained consistent, the rate of interface fatigue crack propagation was nearly identical. The assessment of interfacial fatigue life could be conducted rapidly. In the case of ductile materials, an approximate selection was made [τ] = 0.6σ_b2_. In this context, the substrate’s physical properties were the sole factor considered.

### 2.3. Material Selection

**Figure 2:**
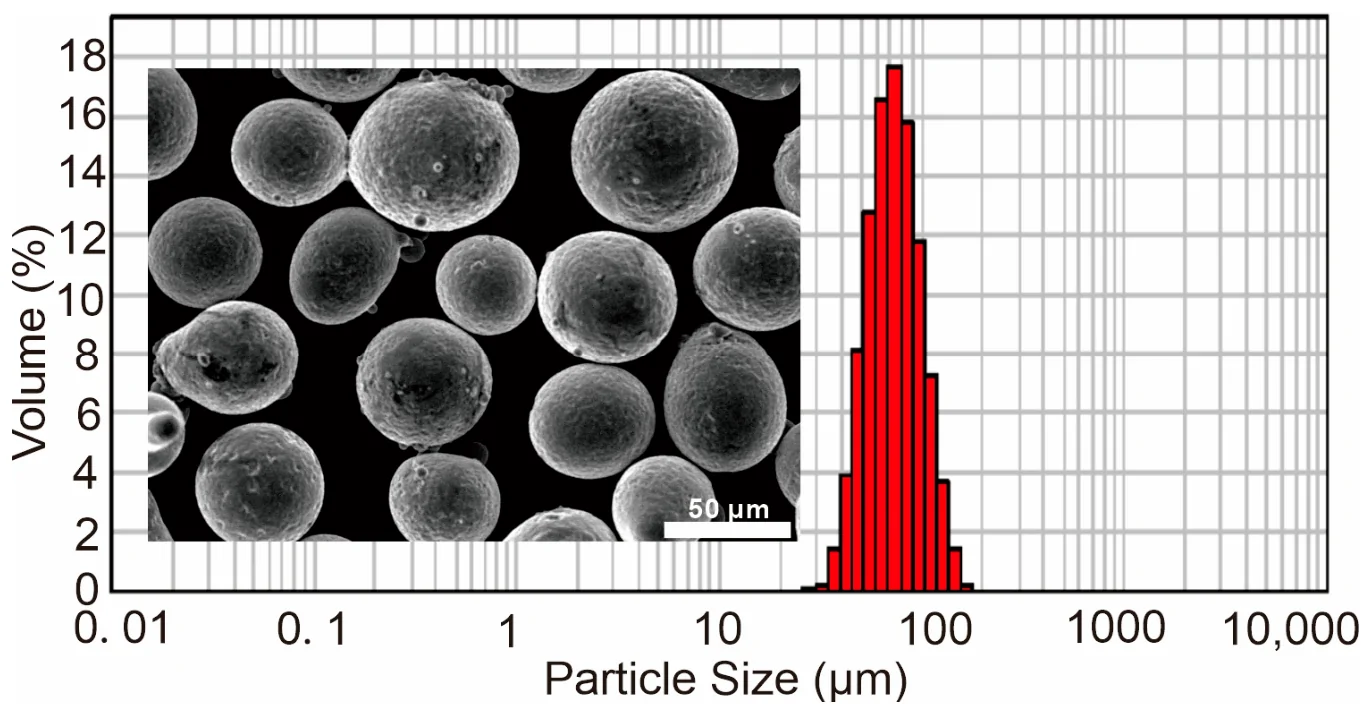
Particle size distribution and morphology of Fe45 and In625 powders.

A nickel-based In625 powder, celebrated for its exceptional wear resistance and featuring a particle size distribution within the range of 15 to 53 μm, was chosen as the raw material. The principal chemical composition is presented in Table 1. The particle size distribution, Hall flow rate (ensuring good flowability), apparent density, and precise control of oxygen content in powder materials significantly influence the mechanical properties of the final products. As illustrated in Figure 2, the median particle size of In 625 powder measures 73.265 μm. To guarantee the mechanical properties of the repaired components, it is imperative to maintain the oxygen content of the powder below 100 PPM. Before the laser cladding process, the powder underwent thermal drying at 60 °C for 8 h to reduce moisture levels. The selection of substrate materials was methodically carried out based on their strength characteristics in comparison to In625, covering three scenarios: materials with enhanced mechanical properties, those with similar strength, and those with lower performance. Furthermore, substrates exhibiting both higher and lower coefficients of thermal expansion (CTE) than In625 were included to assess their thermo-mechanical compatibility. The final selection of substrates included six engineering materials: ASTM 250S (gray cast iron), 1045 medium-carbon steel, 5140 low-alloy structural steel, AISI 304 austenitic stainless steel, GH3625 (a reference nickel-based superalloy), and AISI 420B martensitic stainless steel. The primary chemical constituents and selection criteria for the substrate were presented in Table 2. All substrates underwent a standardized surface preparation process, which involved mechanical polishing with progressively finer SiC abrasive papers followed by ultrasonic cleaning in acetone to ensure cleanliness. Pre-cracked three-point bending tests were conducted for interfacial fracture analysis on the first five materials, while the last five were evaluated for dry rolling/sliding contact in coating–substrate systems. A summary of the key thermophysical properties of the selected materials is presented in Table 3.

### 2.4. Preparation of Laser Cladding In625 Coatings

**Figure 3:**
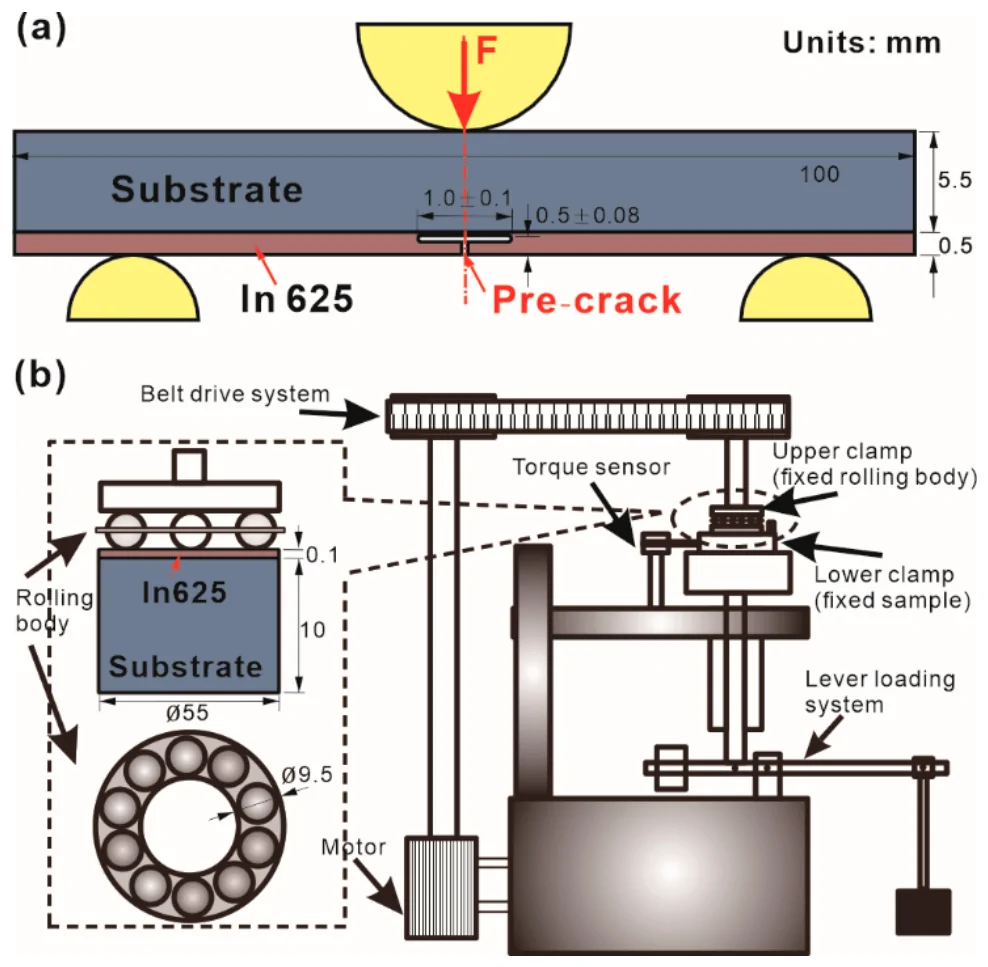
The pre-crack three-point-bending experiment (**a**) and schematic diagram of rolling contact fatigue testing machine (**b**).

YLS-4000 commercial fiber laser system was utilized to create In625 coatings. The primary parameters for the laser process included a linear light source with a power output of 2.8 kW and dimensions of 15 × 1.5 mm. The deposited layer thickness was set to 0.5 mm, with an overlap width of 5 mm and a scanning speed of 10 mm/s. The powder feeding rate was maintained at 20 g/min, while the shielding-gas flow rate was set to 20 L/min. The oxygen concentration in the protective atmosphere was controlled below 500 ppm throughout the deposition process. To ensure compliance with the size specifications of the relevant equipment, all samples underwent wire cutting, surface grinding, and coarse sanding. The dimensions for the three-point bending samples were 100 × 6.0 × 6.0 mm, with the In625 layer measuring 0.5 mm thick. A symmetrical crack, measuring 0.5 ± 0.08 mm in depth and 1.0 ± 0.1 mm in length, was intentionally created near the center of the interface, as illustrated in Figure 3a. For the rolling contact fatigue samples, the overall size was 55 × 10.1 mm, with the In625 layer at 0.1 mm thick, as depicted in Figure 3b. Following grinding and polishing, the roughness of the contact surface was approximately 35 μm (Ra).

### 2.5. Mechanical Experimentation

The strength of the interface is an intrinsic characteristic of the material at the interface phase, indicating that failure due to cracking is fundamentally similar to failure occurring in the absence of cracks. This principle underpins the pre-crack three-point bending tests. These bending tests were conducted at ambient temperature using an Instron-5500R universal testing apparatus (Instron Corporation, Wuhan, China). The indenter was positioned on the substrate side, while the two supports were placed on the In625 side to ensure that the interface beneath the neutral axis experienced tension. The parameters for the experiment included an indenter diameter of 15 mm, support diameters of 30 mm, a span of 50 mm, and a loading rate of 0.2 mm/min. Each group underwent three tests, and the results were averaged for statistical analysis.

A rolling contact machine featuring a ball-on-disk configuration was employed to assess the rolling contact fatigue (dry rolling/sliding contact) characteristics of laser-clad In625 coatings. The setup included a bearing with ten evenly spaced balls, which served as the friction pair. These balls, made from AISI 52100 steel, had a diameter of 9.5 mm, an average surface roughness of 0.012 μm, and a Rockwell C hardness of 60 HRC (with elastic modulus ***E*** = 219 GPa and Poisson’s ratio ***υ*** = 0.26). A synchronous motor regulated the shaft’s rotational speed, maintaining it consistently at 1200 rpm. A loading rod applied a force of 2450 N to the lower section using a lever arm, ensuring that any interface failure was attributed to stress fatigue. The experiments concluded under two conditions: reaching a predetermined duration of 1000 min or when the torque signal surpassed a set limit of 5 N/m. All tests were performed at ambient temperature without lubrication, and a new bearing was utilized for each trial. Each group underwent three tests, with the average results calculated for analysis.

An analysis of fracture morphology was carried out through a multi-scale characterization method. The macroscopic failure characteristics observed from static bending and rolling contact fatigue tests were investigated using optical microscopy (OM, Keyence Co., Ltd., Shanghai, China). Three-dimensional surface measurements were obtained with a white-light interferometer (MFT-5000, Rtec Instruments, San Jose, CA, USA). For microstructural analysis, cross-sectional examinations of In625 coatings were performed using scanning electron microscopy (SEM, JSM-IT300, Tokyo, Japan), and high-resolution images of the interfacial regions beneath wear scars were captured using field-emission SEM (FE-SEM, Zeiss Ultra Plus-43-13, Oberkochen, Germany).

## 3. Results

### 3.1. Experimental Results

**Figure 4:**
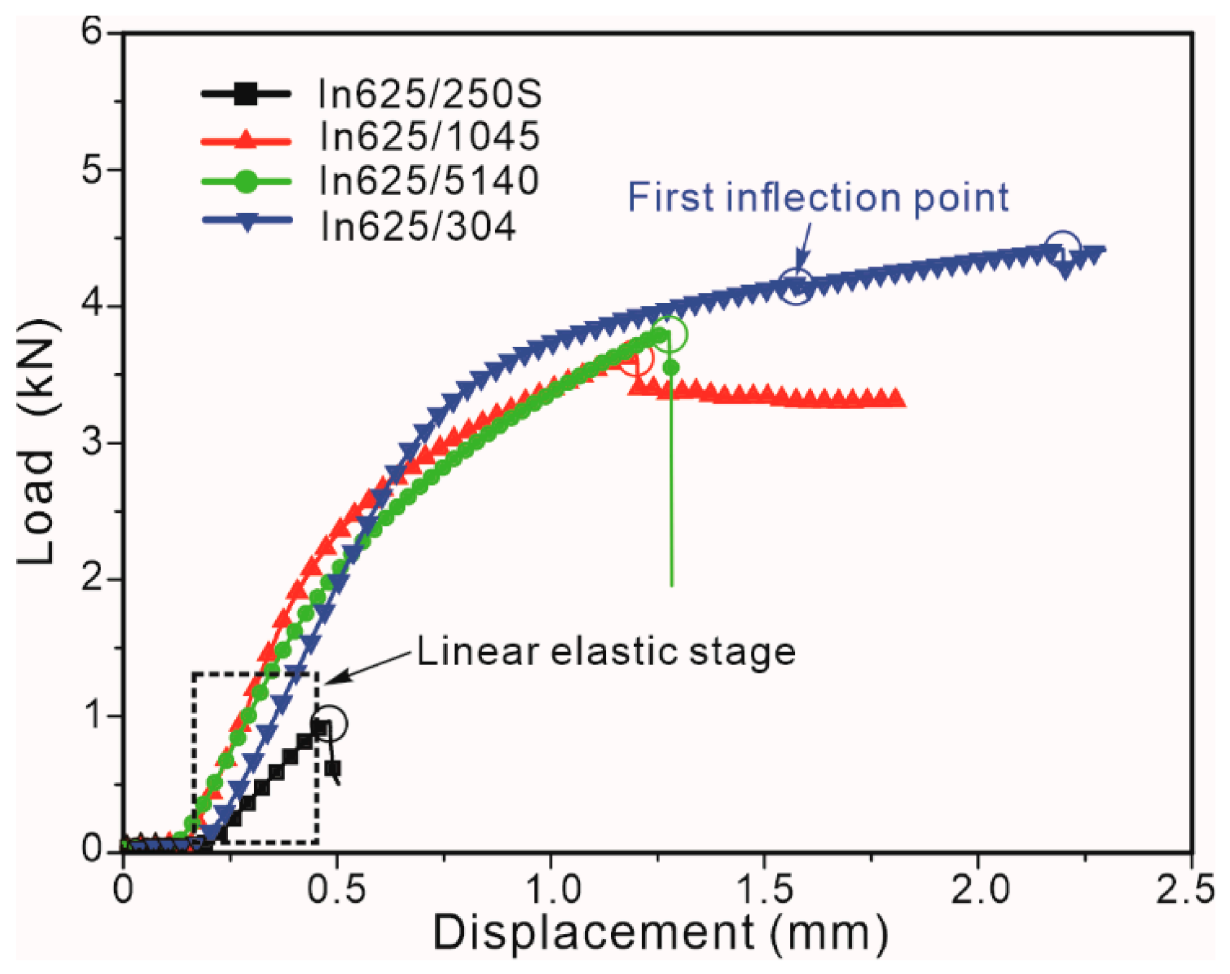
The displacement–load curves under three-point-bending.

The three-point bending experiments conducted on pre-cracked samples clearly demonstrated the significant impact of substrate choice on the mechanisms of interfacial failure, as illustrated in Figure 4. Various fracture patterns were observed:

- Brittle fracture (In625/250S)

Demonstrated linear elastic behavior constrained to strains below 0.5%. A sudden failure was observed at a stress of $\tilde{\tau_{C}}$ = 25.7 ± 1.2 MPa, with no occurrence of plastic deformation.

- Ductile fracture (In625/304)

Exhibited exceptional results with: peak nominal strength of 115.0 MPa, maximum critical displacement measuring 1.54 mm, post-yield strain hardening characterized by n = 0.18 ± 0.02, and continued load-bearing ability surpassing the yield threshold.

- Unstable fracture (In625/1045 and In625/5140)

Exhibited transitional behavior: rapid crack propagation, sharp load drops, intermediate strength (99.2–105.3 MPa).

Table 4 outlines the hierarchy of interfacial strength based on three essential metrics. The nominal interface strength ranks from highest to lowest as follows: In625/304, In625/5140, In625/1045, and In625/250S. The findings reveal a significant relationship (R^2^ = 0.92) between critical displacement and nominal strength.

**Figure 5:**
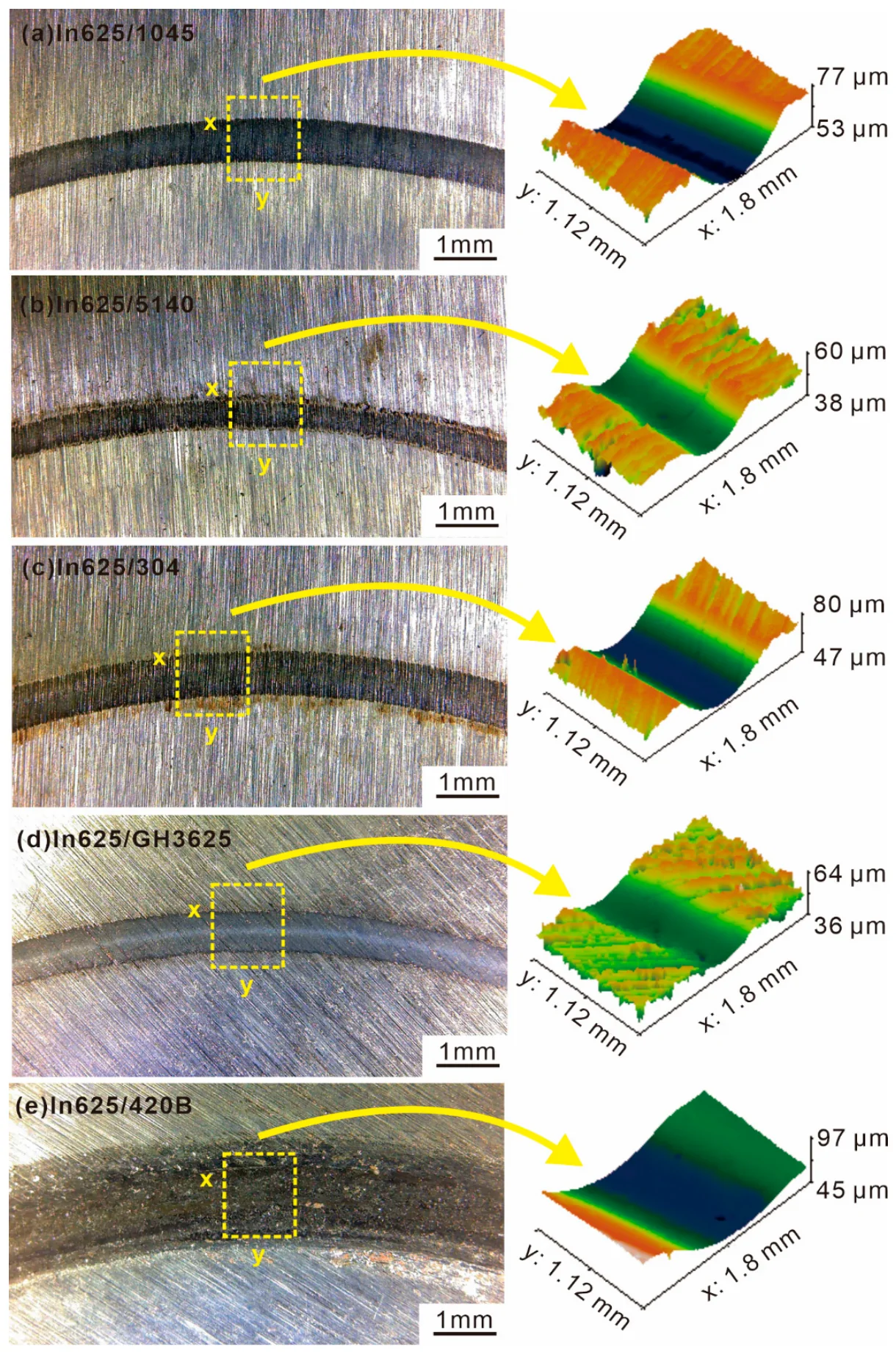
The wear morphology of the In625 coating under different substrates. (**a**) In625/1045; (**b**) In625/5140; (**c**) In625/304; (**d**) In625/GH3625; (**e**) In625/420B.

**Figure 6:**
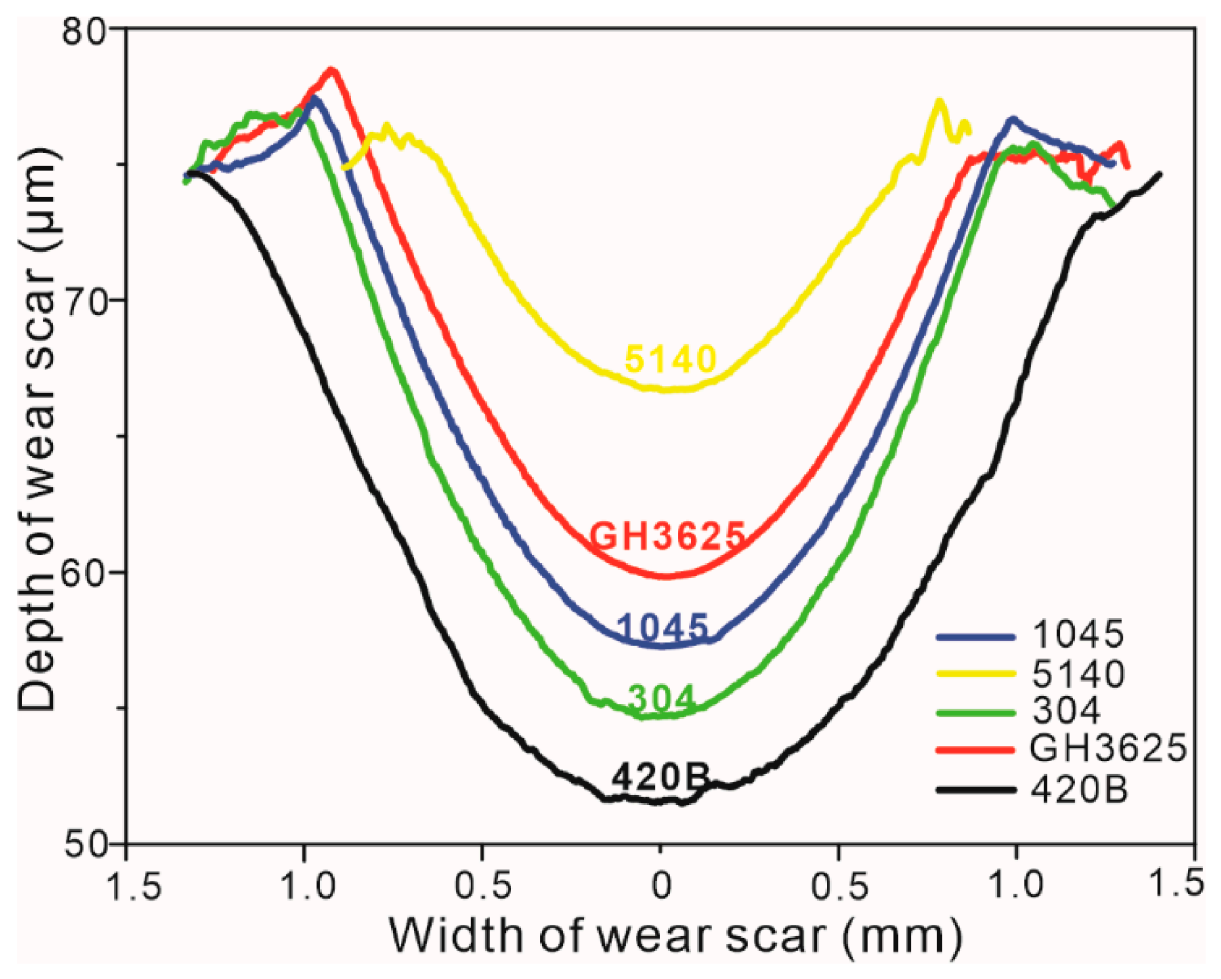
The cross-sectional profile of wear scars.

Analysis of the three-dimensional shape of the surface wear scars indicated notable failure modes that depend on the substrate, as illustrated in Figure 5 and Figure 6.

- Catastrophic wear (In625/420B)

Displayed peak wear measurements: Width: 1.31 ± 0.05 mm (beyond the microscope’s field of view). Depth: 23.2 ± 1.1 μm. The presence of significant macroscopic debris suggested intense adhesive wear. Initial failure occurred at 4.4 × 106 cycles, which is 56% below the average.

Primary failure mechanism: Thermal expansion mismatch and Residual stress concentration.

- Optimal performers (In625/5140 and In625/GH3625)

Negligible surface impairment: Depth of wear is less than 15 μm. Reduction in width can reach 30% compared to 420B. Absence of plastic deformation at the edges indicates a purely fatigue-driven process.

The quantitative evaluation of fatigue highlighted a clear ranking among the various coating–substrate pairs (as shown in Table 5): In625/5140 > In625/GH3625 > In625/1045 > In625/304 > In625/420B. This ranking reveals two significant discrepancies that challenge traditional material selection guidelines: the Strength-Fatigue Life Paradox and the Chemical Similarity Trap. Notably, the 420B substrate demonstrated a fatigue life that was 56% shorter compared to the 304 substrate, indicating a breakdown of classical strength theories in the context of interfacial fatigue. Although GH3625 has a chemical composition most similar to IN625, its fatigue performance was still 18% less effective than that of the best-performing In625/5140 combination. The maximum subsurface stress and shear-stress distribution may differ from those predicted for a homogeneous material. The coating thickness and the mechanical mismatch between the coating and substrate can modify the location and magnitude of the critical stress, as well as the relative susceptibility of the coating, interface, and substrate to crack initiation.

### 3.2. Model Validation

Based on the findings presented in Table 4, the interfacial shear strength model for the In625 coating is defined in Equation (13). To validate this model, the In625/GH3625 combination was utilized. Table 6 illustrates the comparison between the calculated results from the model and the outcomes from three-point bending experiments, demonstrating that the model effectively assesses the heterogeneous interface strength of In625 coatings.

(13) $$\tilde{\tau_{C}}=0.5834\tau_{1s}+0.0745\tau_{2s}-0.0127\left(\alpha_{1}-\alpha_{2}\right)T_{1}\frac{\sigma_{1b}}{\sigma_{1s}}+\;0.009\left(\alpha_{1}-\alpha_{2}\right)T_{2}\frac{\sigma_{2b}}{\sigma_{2s}}$$

**Figure 7:**
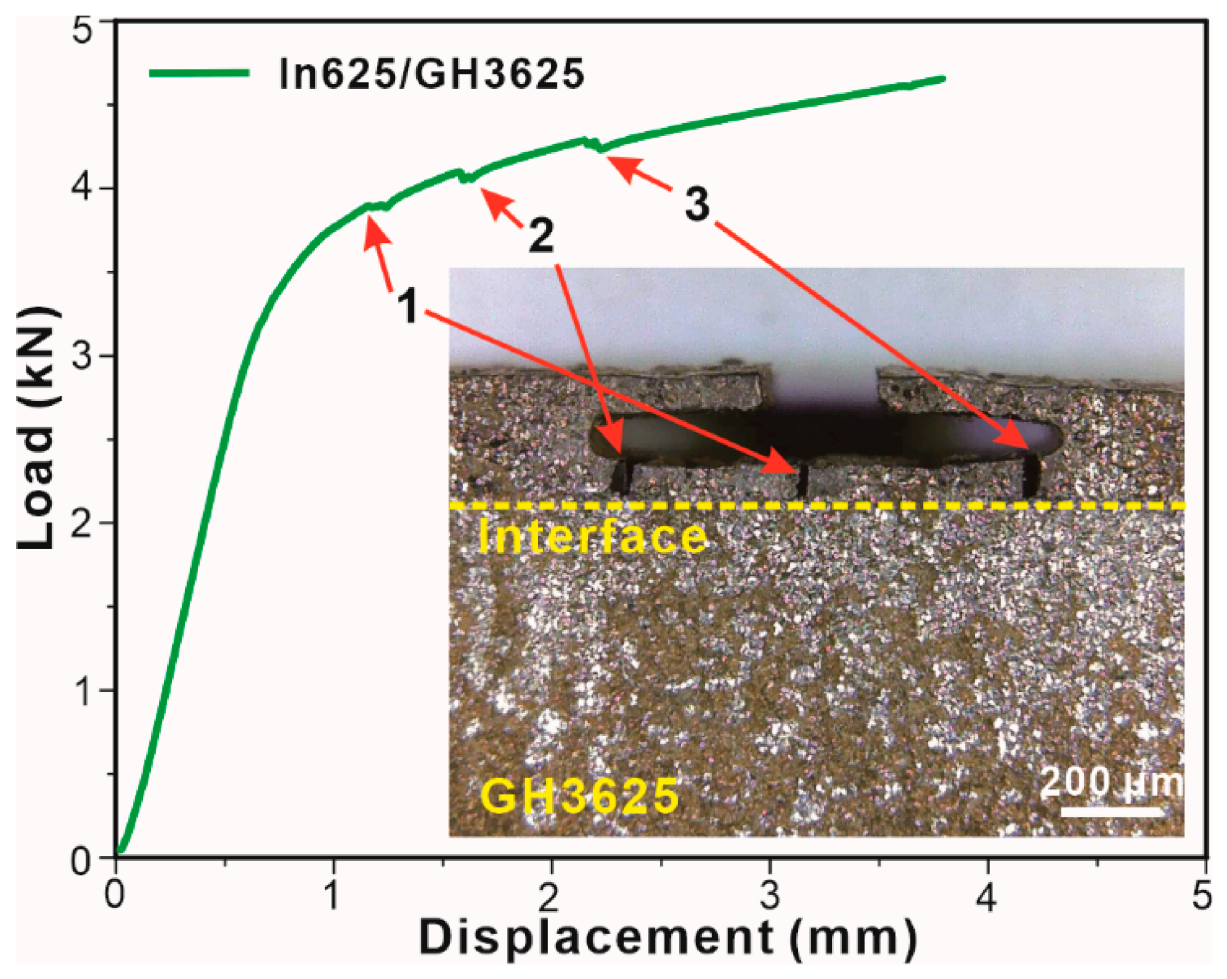
Displacement–load curve and failure location of In625/GH3625 under bending load.

Figure 7 shows that the displacement–load curve for In625/GH3625 features three notable inflection points, each representing successive changes in stiffness and damage state. The initial fracture at Position 1 follows a path that aligns with the direction of maximum shear stress, whereas Positions 2 and 3 reveal localized failure near the pre-crack tip area, suggesting that the interfacial failure mechanisms are influenced by the material properties.

**Figure 8:**
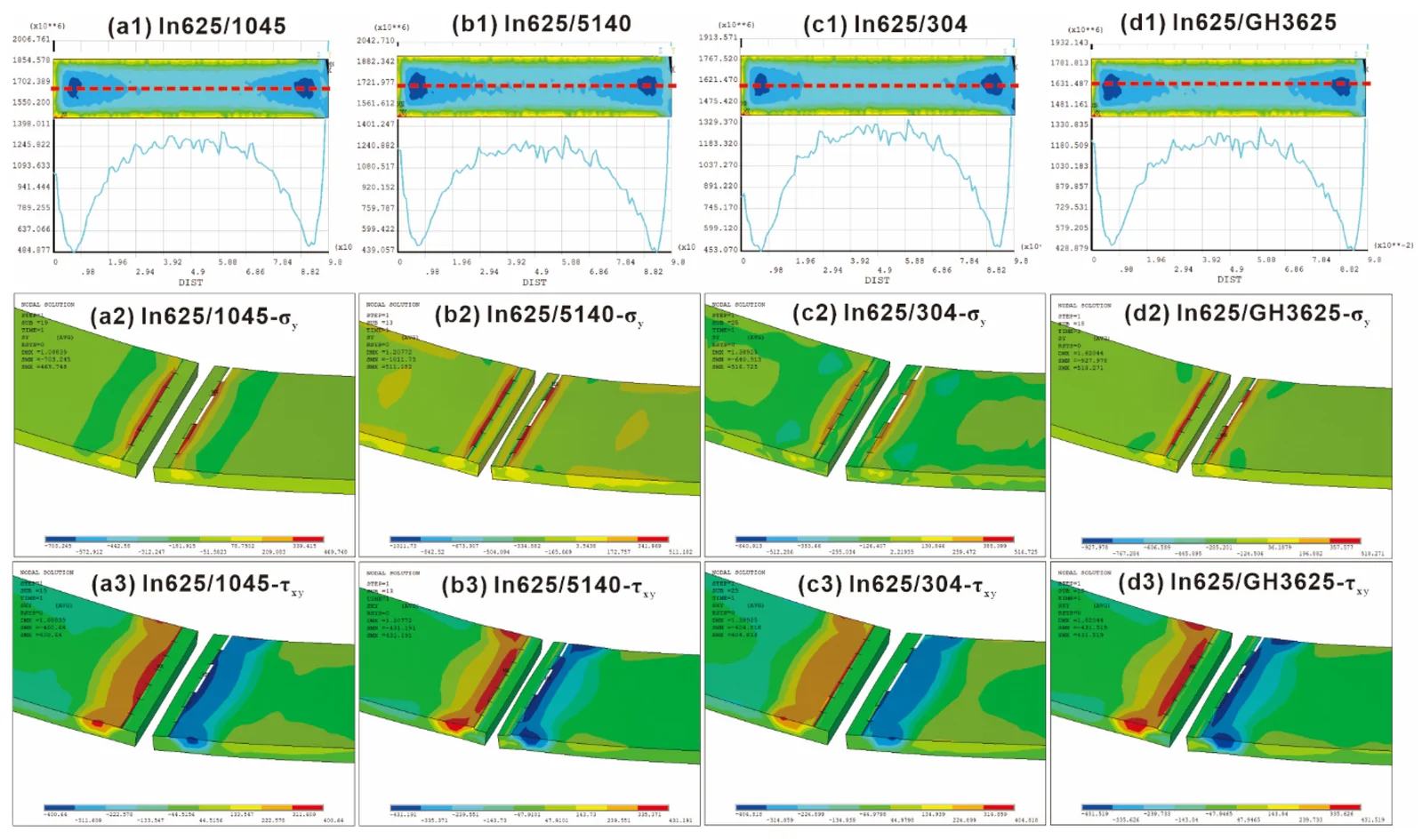
Thermal residual stress analysis of In625/substrates (1045/5140/304/GH3625) at the interface midline (**a1**–**d1**). Critical failure normal (**a2**–**d2**)/shear stress (**a3**–**d3**) at the pre-crack tip under bending load.

To ascertain ***K*_2*C*_**, a finite element analysis was utilized to measure the distributions of normal and shear stresses at the interface during three-point bending (see Figure 8). The calculated stress field and the predicted critical damage location depend on the coating thickness, the material–property mismatch, and the interfacial strength. Characterization following laser cladding indicates that the buildup of thermal residual stress primarily influences the modes of coating failure. The stress field at the interface shows a significant increase in stress near the contour (15–20% greater than in the central area), a bimodal distribution with lower stress levels near the start and end zones, and a consistently high stress plateau at the location prior to crack formation.

Stress profiling along the interface centerline (dashed red) demonstrates that mismatch in:

- Yield strength (***σ_s_***)
- Thermal expansion coefficients (α)

are key factors contributing to variations in interfacial stress. The essential failure scenario was examined by combining residual stress and bending load distributions, while disregarding stress relaxation caused by pre-existing cracks for the sake of simplifying the model. Significant aspects include:

- Symmetric normal stress distribution (σ_yc_);
- Antiphase shear stress pairing (τ_xyc_).

The critical failure criterion was established based on the maximum stress observed on the left side. An analysis comparing various material combinations (In625/1045, In625/5140, In625/304, In625/GH3625) indicates that the pairing with GH3625 provides the best stress redistribution (see Table 7), demonstrating an 18% greater tolerance for normal stress compared to its 1045 medium-carbon steel equivalent.

**Figure 9:**
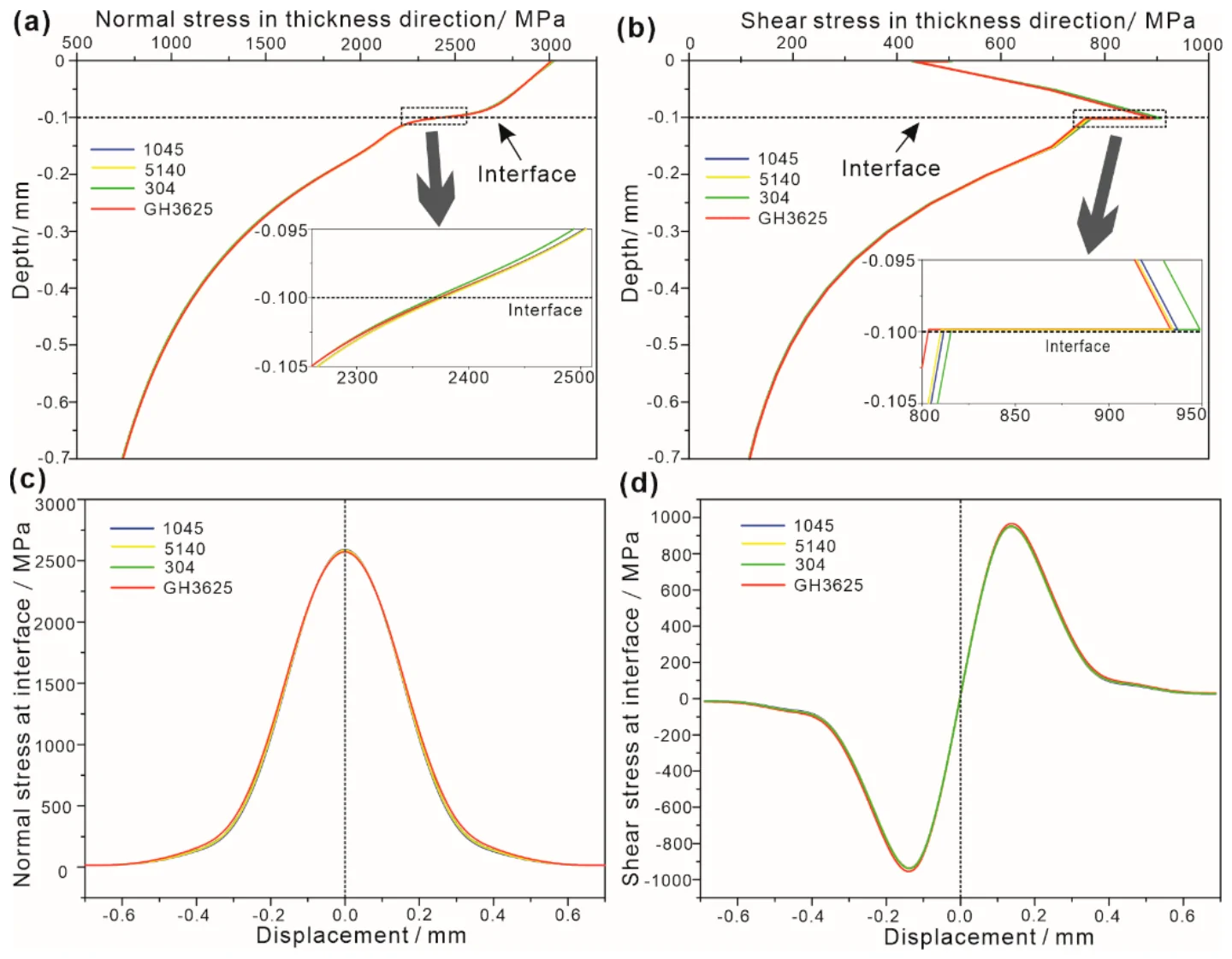
The distribution characteristics of normal/shear stress along thickness (**a**,**b**) and interface (**c**,**d**) under rolling contact condition.

The factors for stress intensity (***K*_1*a*_**/***K*_2*a*_**) were calculated using Hertzian contact analysis, focusing on essential parameters such as contact radius (a = 0.197 mm), peak contact stress (3.079 GPa), and maximum shear stress (τ_max_ = 0.98 GPa) found at a subsurface depth of z = 93 μm. For detailed derivations of the relevant content, please refer to Appendix A. A 2D axisymmetric finite element model was utilized, incorporating boundary conditions at the base of the specimen and along the contact axis (refer to Figure 9a,b). The outcomes of the numerical analysis indicated:

- Fatigue failure mechanism

The concentration of stress at the interface facilitated the initiation of cracks at that site, which then extended in the direction of the maximum principal stress (see Figure 9c).

- Stress gradient evolution

The normal stress showed an exponential reduction through the thickness, starting at 2.5 GPa at the surface and dropping to approximately 0.5 GPa at a depth of 200 μm. Meanwhile, the shear stress reached its maximum of 900 MPa close to the interface of the two materials (see Figure 9d), aligning with a principal stress plane at 45°.

- Substrate independence

Statistical evaluation revealed that the variations in **σ_z_**/***τ_xy_*** distributions among different substrate materials were less than 5%, suggesting that the primary influence on the contact response was the properties of the coating.

Based on the derivation presented, Table 7 lists the calculated values for the parameter ***C***, critical damage ***D_C_***, and the equivalent shear strength [**τ_C_**] for all samples. The model determined the ranking of interface fatigue characteristics as follows: 5140 > GH3625 > 1045 > 304 > 420B. The findings from the model’s derivation align perfectly with the experimental outcomes regarding the interfacial equivalent shear stress at critical failure. It is important to note that this finite element analysis model primarily accounts for normal contact loads and heterogeneous coating/substrate configurations. Given that the friction coefficient was not measured in this experimental procedure, the tangential traction force, which relies on friction, was not independently calibrated. Consequently, the computed orthogonal shear stress represents the stress field under the assumptions of the employed model and should not be interpreted as a universal stress distribution applicable to all lubricated bearing contact scenarios.

## 4. Discussion

### 4.1. Metallographic Analysis of Surface and Heterogeneous Interface

Figure 9 illustrates the distinct microstructure of the laser-clad In625 alloy, which consists of a γ-Ni matrix interspersed with precipitates (average distance: 1.2 ± 0.3 μm) and corrosion voids (size: 0.5–2 μm). The sequence of solidification is as follows: L → L + γ (with a transition temperature of 1360 °C) → L + γ + MC (at 1240 °C) → L + γ + MC + Laves (at 1120 °C) → γ + MC + Laves (at ambient temperature) [25]. Notable microstructural characteristics include:

- Primary γ-phase: FCC structure (a = 3.592 Å) with limited solute partitioning (Cr < 15 at.%, Nb < 2 at.%);
- MC carbides: Nb-rich with cubic morphology at grain boundaries;
- Laves phase: Hexagonal A_2_B-type.

**Figure 10:**
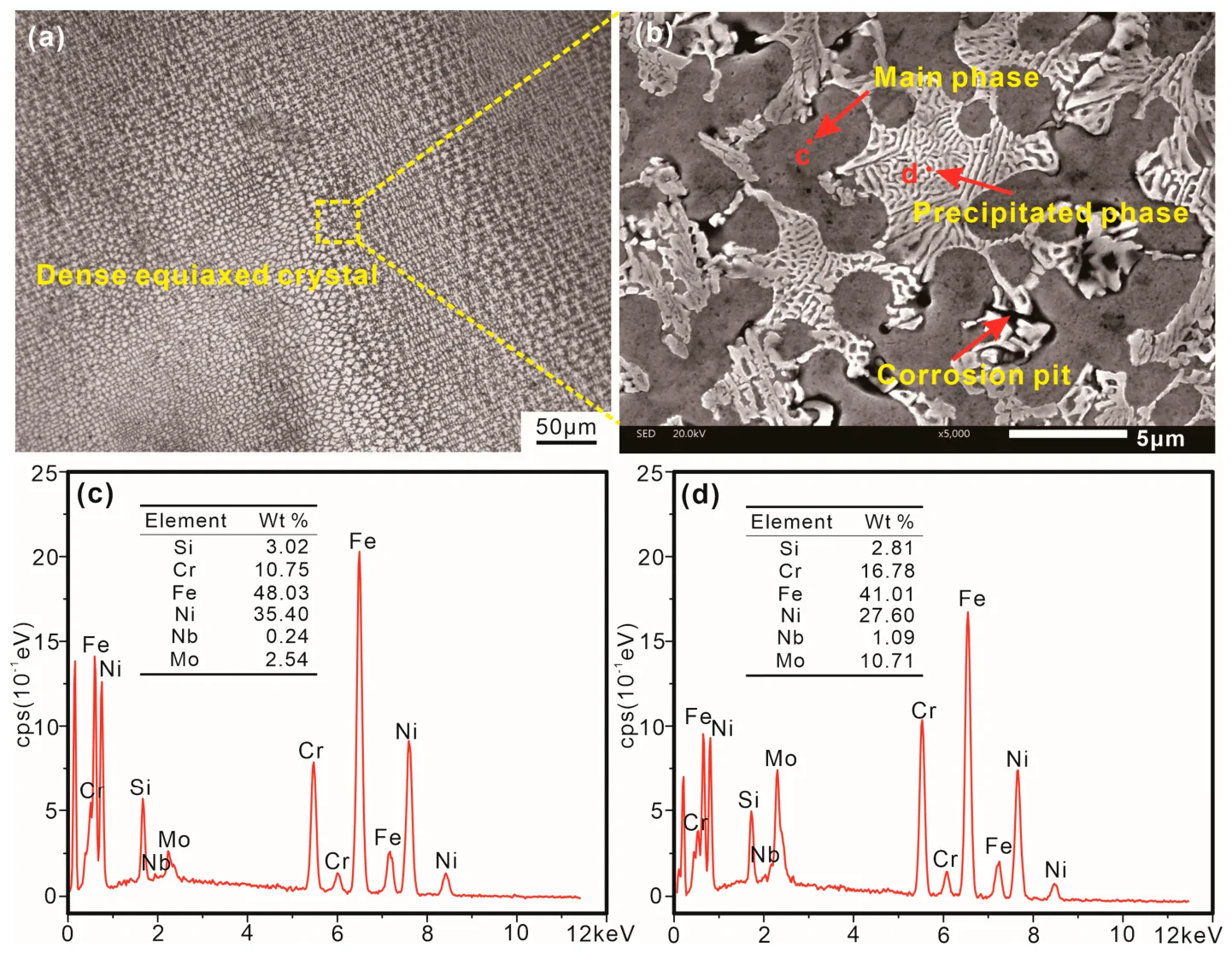
Metallographic and Energy Spectrum Analysis of In625 Coatings. (**a**) SEM (×250); (**b**) SEM (×5000); (**c**) main phase; (**d**) precipitated phase.

EDS analysis revealed that the primary phase γ-Ni was rich in Fe, Ni, and Cr, along with trace amounts of Nb and Mo, as illustrated in Figure 10c. In contrast to the substrate, the network precipitates (TCP phase, Point d) exhibited lower levels of Fe and Ni, while the concentrations of Mo, Nb, and Cr were markedly elevated. Notably, the σ phase had a high concentration of Cr, whereas the μ and P phases showed elevated levels of Mo. The development of these phases necessitated the segregation of Nb, Mo, and Cr, leading to their reduced presence in the substrate, which aligned with the findings from the energy spectrum analysis.

**Figure 11:**
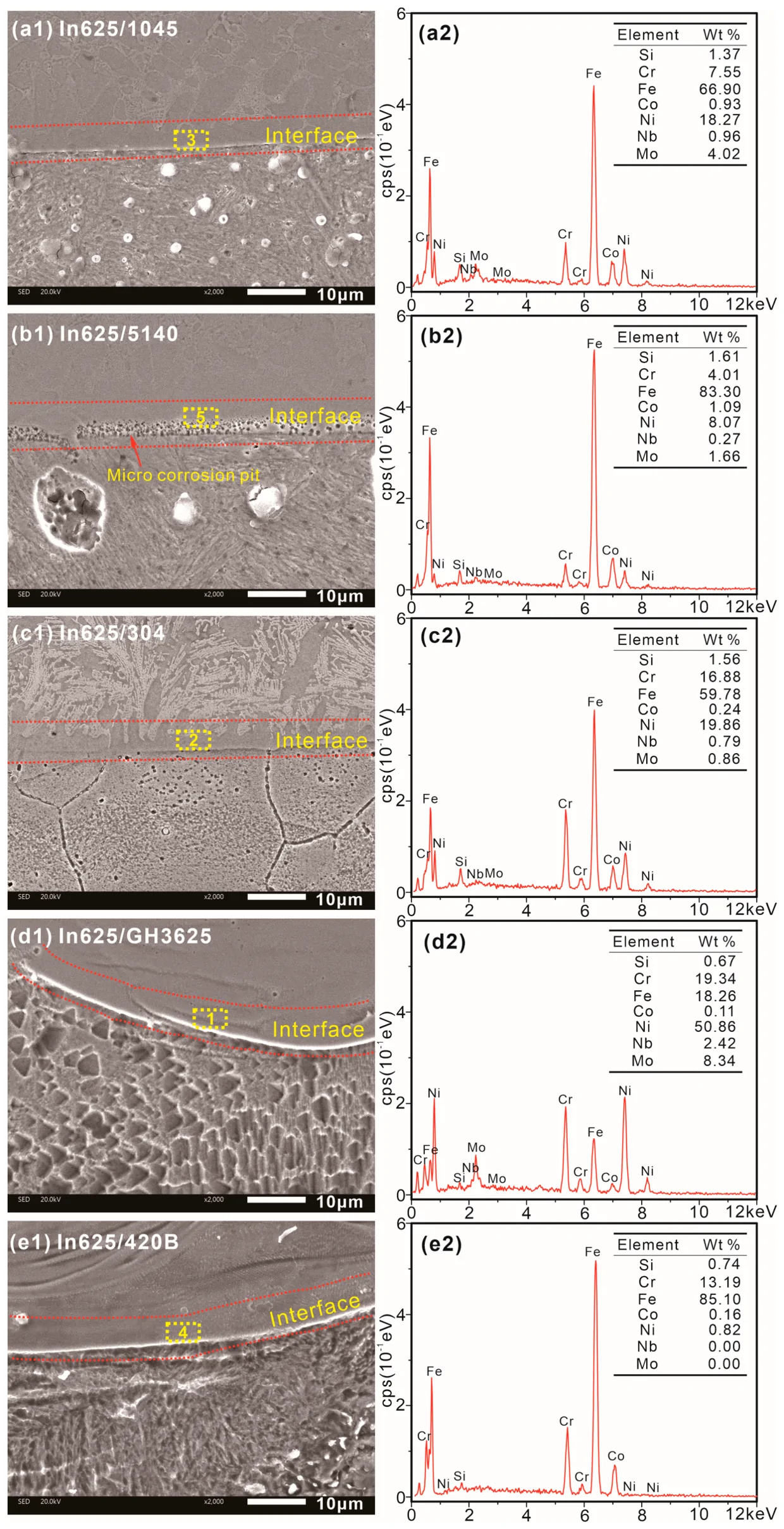
Metallographic and EDS analysis of interface region under different material matching. (**a**) In625/1045; (**b**) In625/5140; (**c**) In625/304; (**d**) In625/GH3625; (**e**) In625/420B.

The analysis of the metallographic structure and the composition at the interface is presented in Figure 11. It was noted that multiple planar crystal bands, each measuring several microns in width, were present between the repair coating and the substrate. A clear distinction in the level of corrosion on either side of the interface was evident, particularly in the In625/5140 pairing (Figure 11b). Numerous corrosion pores were observed at the interface adjacent to the substrate (Figure 11e). Among the five sample groups, the concentration of the Cr element was found to be the lowest.

### 4.2. Fracture Analysis

**Figure 12:**
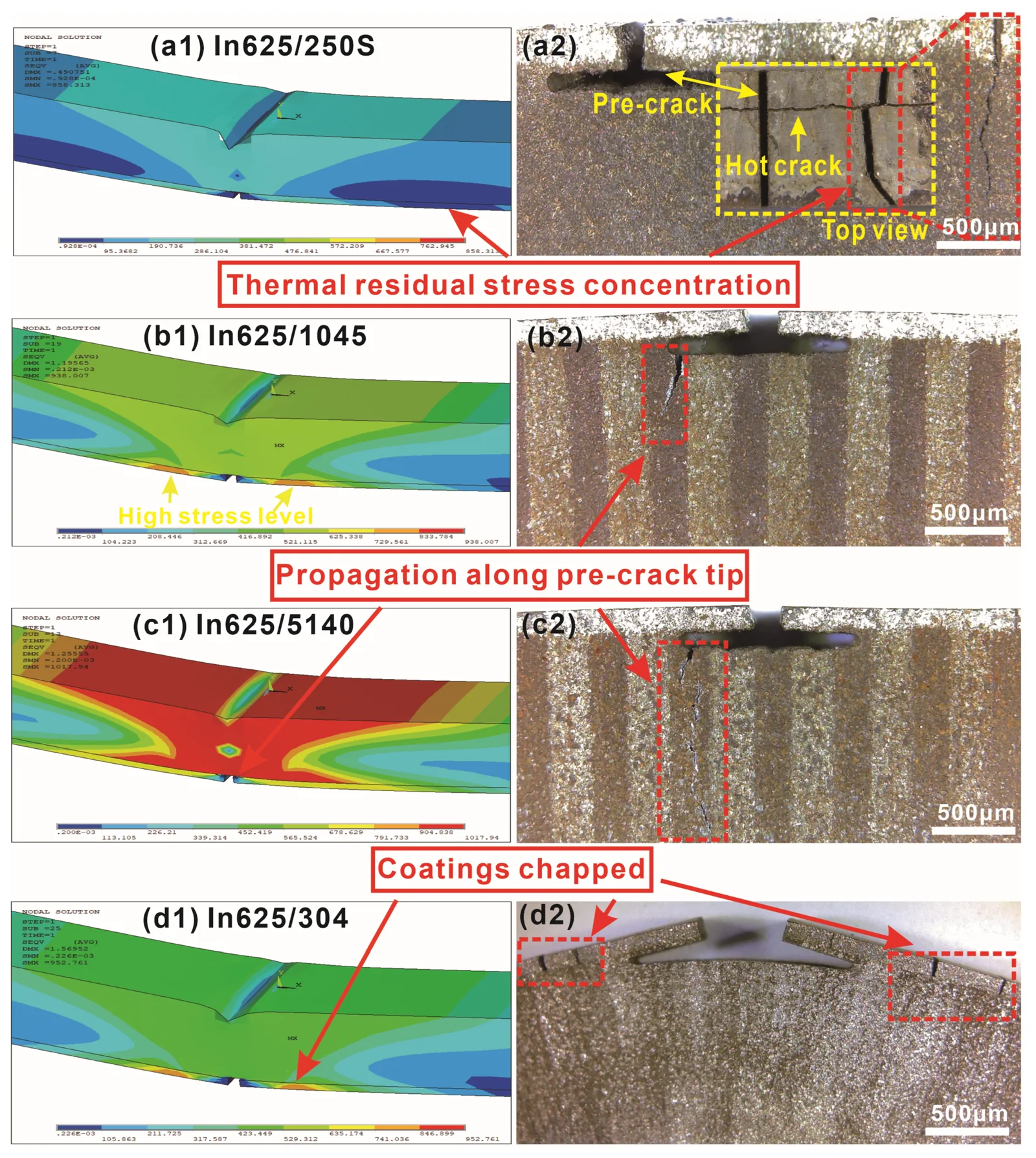
Comparison of three-point-bending failure modes of different materials matching. (**a**) In625/250S; (**b**) In625/1045; (**c**) In625/5140; (**d**) In625/304.

Figure 12 illustrates a comparison of the fracture behavior under static load, contrasting numerical simulations on the left with experimental results on the right. The varying substrate compatibility at the heterogeneous interface significantly influenced the failure modes of the In625 coating. Three primary failure modes were observed for the In625 coating when subjected to static loading in a three-point bending test.

(1) In625/250S system: thermal crack failure by residual stress accumulation

Quantitative forecasting indicates minimal substrate stress (approximately 320 MPa) accompanied by significant plastic deformation near the indenter (ε_p_ > 35%). There is a 18% decrease in load-bearing capacity when compared to a model without stress.

(2) In625/1045 and In625/5140 systems: propagation along interfacial pre-crack tip

Due to the significantly greater thickness of the substrate compared to the In625 coating, the load-bearing ability of the composite component was primarily reliant on the substrate itself, resulting in a variation in stress distribution between the two specimens.

(3) In625/304 system: coating chapped failure

Using the critical failure displacement of In625/GH3625 (1.21 mm as shown in Figure 7) as a reference point—given that GH3625 shares similar composition and characteristics with In625—it is evident that the plasticity of In625/304 significantly exceeds that of In625/GH3625.

**Figure 13:**
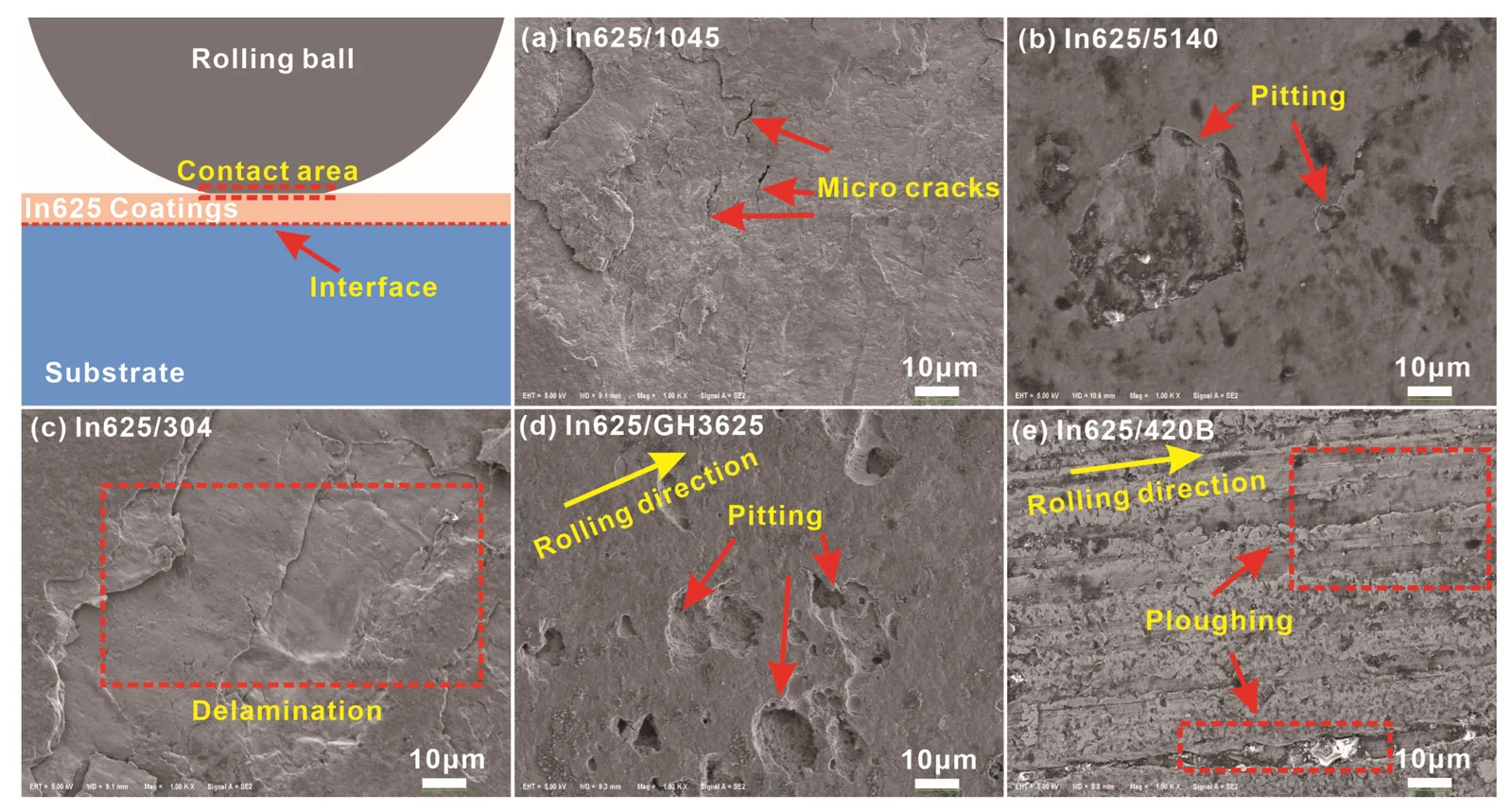
Comparison of rolling contact fatigue failure surfaces under different substrates. (**a**) In625/1045; (**b**) In625/5140; (**c**) In625/304; (**d**) In625/GH3625; (**e**) In625/420B.

Figure 13 illustrates the analysis of wear surface morphology in the context of rolling contact fatigue. The In625/420B sample uniquely activated the sensor threshold after 367 min, whereas the other samples finished their tests at set times without reaching this threshold. Clear ploughing marks oriented with the rolling direction were noted, along with considerable material delamination. In the absence of lubrication during dry rolling/sliding contact test, wear debris built up in the contact tracks due to insufficient removal. Significantly, shiny white abrasive particles found in the ploughing pits intensified surface deterioration through three-body abrasion processes.

The choice of substrate material had a notable impact on the failure mechanisms observed in In625 repair layers. The interface between In625 and 5140 primarily displayed pitting corrosion characteristics (see Figure 13b), indicating a relatively low level of wear when compared to other samples. These micro-pits, which typically arise from surface micro-slip and contact between asperities during sliding, signify early-stage surface damage. A quantitative assessment showed that the micropores caused by pitting were significantly smaller than the delamination features, resulting in insufficient vibrations to trigger the torque sensor’s threshold. Cross-sectional analysis (refer to Table 5) revealed a maximum wear depth of 23.2 μm, which is less than the thickness of the In625 layer, and there was no indication of crack propagation at the interface leading to extensive spalling. This implies that the strength of the interface, influenced by the substrate, has a limited effect on the selection of surface failure modes.

The coating thickness is an important parameter in determining the rolling-contact response of a coated raceway. The relevant thickness is not only the absolute thickness of the deposited layer, but also its ratio to the contact radius and to the depth of the stress-affected subsurface region. When the coating is sufficiently thick relative to the affected depth, the near-surface stress field may be governed primarily by the coating. In contrast, when the coating is relatively thin, deformation in the substrate can strongly influence the stress distribution within the coating and near the coating/substrate interface. This condition is commonly associated with the eggshell effect in coated rolling-contact systems. From a fracture-mechanics perspective, reducing the coating thickness does not necessarily lead to a proportional reduction in fatigue performance. A thin coating may reduce material consumption and facilitate remanufacturing, but the substrate constraint and the resulting interfacial shear stress may become increasingly important. In the present study, the coating thickness was fixed at and was therefore not treated as an independent experimental variable. The present results should consequently be interpreted as a comparison of different substrate materials under a fixed coating architecture. We have not claimed a universal thickness threshold for bearing fatigue life.

**Figure 14:**
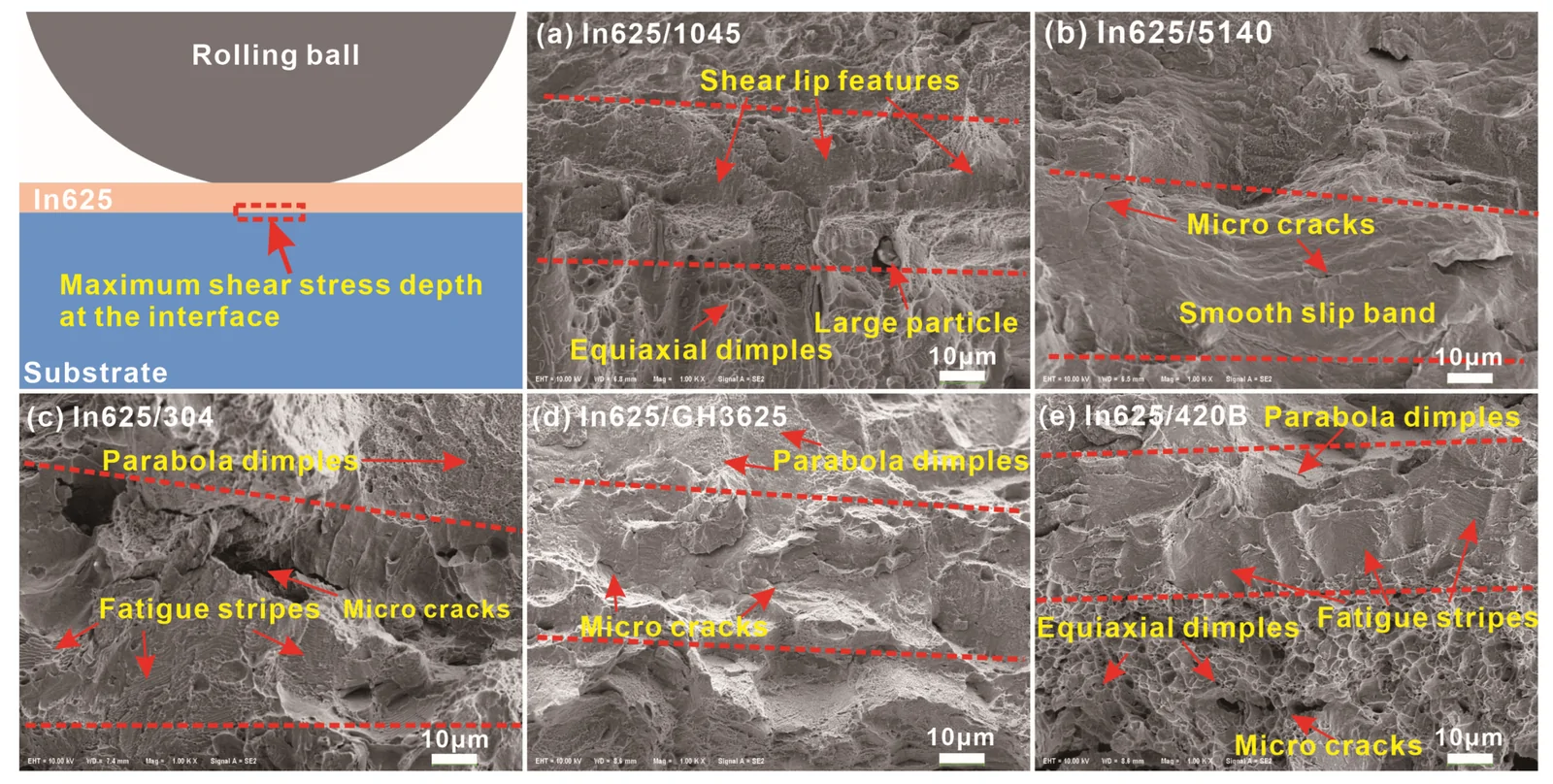
Comparison of interface fracture under different substrates. (**a**) In625/1045; (**b**) In625/5140; (**c**) In625/304; (**d**) In625/GH3625; (**e**) In625/420B.

The thermal characteristics of the substrate, including its density, thermal expansion coefficient, specific heat, conductivity, and convection coefficients, play a crucial role in shaping the thermal gradients during the laser repair process. This, in turn, influences the residual stress conditions in In625 deposits and ultimately dictates the modes of surface failure. The observed fracture characteristics should be understood as the combined result of repeated normal contact loading, friction-induced tangential traction, and stress concentration caused by the heterogeneous coating/substrate interface. The interfacial fatigue markings and advancing microcracks support the occurrence of cyclic crack growth; however, the dry condition may also enhance surface-related frictional damage. Theoretical evaluations suggest that the varied interface is located in the area where cyclic shear stress peaks (see Figure 14). Clear slip bands can be seen in the phase transition area outlined by the red dotted lines. Importantly, cracks and fatigue striations aligned with the shear direction are visible in this area, especially prominent in the In625/420B sample.

In In625/GH3625, the interface shows signs of slipping and minor microcracks, yet the structure of the interfacial phase is similar to that of unadulterated In625 coatings.

In sample In625/304, notable destruction is evident above the bonding junction, marked by stretched parabolic indentations. The presence of advancing microcracks and distinct fatigue markings suggests a considerable amount of dislocation activity at the interface, resulting from the interplay of normal and shear stresses.

In the fracture morphology observed at In625/1045, equiaxed dimples are primarily found beneath the interface. The slip band at the interface shows distinct fatigue striations, and the microstructure situated above the interface also reveals equiaxed dimples. Notably, sizable unmelted particles can be seen close to the interface in the substrate, with fatigue cracks extending towards these particles.

In the interfacial phase of In625/5140, there are highly polished shear bands that feature a multitude of slender, elongated fatigue fractures.

Although both the In625 coatings and their substrates possess excellent fracture toughness, the uneven interface displayed traits of brittle fatigue failure. When subjected to significant shear stress and localized plastic deformation, the fatigue damage advanced until it met the conditions for static failure, leading to the formation of cracks that eventually resulted in the failure of the coating.

The test matrix for bending and rolling contact has not been fully cross-validated. Specifically, In625/250S was subjected solely to bending tests, without undergoing rolling contact tests; conversely, In625/420B underwent only rolling contact tests, without corresponding independent bending measurements. Consequently, the predictions for In625/420B should be regarded as model-based inferences or partial verifications, rather than as a fully independent validation of interfacial strength. According to the statistical evaluation of the measurements for wear scar width and depth shown in Table 5, the ranking of fatigue performance is as follows: 5140 surpasses GH3625, which in turn is better than 1045, followed by 304, and lastly 420B. This order of performance is directly linked to the morphological features observed in the interfacial slip bands among the different material combinations.

## 5. Conclusions

The present study demonstrates the influence of heterogeneous coating/substrate interfaces on cyclic rolling/sliding contact damage and interfacial fracture in laser-remanufactured In625 coatings under the adopted dry-contact condition. The physics-based interfacial-strength model and equivalent-shear-stress criterion provide a comparative framework for evaluating the five investigated substrate systems. It is important to note that this study elucidates how substrate selection and heterogeneous interface characteristics influence the cyclic rolling/sliding contact damage observed in laser-reprocessed In625 coatings under the specific laboratory conditions utilized, and it does not aim to offer a direct predictive approach for the dry rolling/sliding contact life of lubricated bearings. The main contribution of the present study is the identification of the role of heterogeneous interfaces in cyclic rolling/sliding contact damage of laser-remanufactured In625 coatings under the specified laboratory conditions.

(1) The mechanical model of the interface demonstrated variations in critical damage states (***D_C_***) that depend on the substrate. These variations were measured using interface stress intensity factors, failure criteria, a model for nominal interfacial shear strength, and a model for the evolution of critical damage.
(2) Three-point bending tests conducted prior to cracking confirmed the shear strength model and revealed three unique modes of static load failure: surface cracking caused by thermal residual stress, propagation of interface cracks, and surface chipping.
(3) Testing of five substrate materials (1045, 5140, 304, GH3625, 420B) for dry rolling/sliding contact validated the accuracy of the model. Analysis of fracture surfaces indicated that the fatigue lifespan is influenced by the interplay between peak surface contact stress and the buildup of shear stress at the interface.

The developed frameworks facilitate the numerical evaluation of interface cyclic rolling-contact damage effectiveness, offering essential information for enhancing remanufacturing processes.

## Figures and Tables

**Table 1:** Chemical composition of powder materials (wt%).

	C	B	Si	Mn	Cr	Ni	Fe	Co	Mo	Nb	Al	Ti
In625	0.04	-	0.13	0.05	20.3	Bal	3.77	0.03	8.89	3.73	0.1	0.24

**Table 2:** Chemical composition of substrate materials (wt%).

Grade	Material Category	Supply Condition	C	Si	Mn	Cr	Ni	Fe	Mo	Nb	Al	Ti
250S	gray cast iron	as-cast	3.16	1.81	0.89	-	-	Bal	-	-	-	-
1045	medium-carbon steel	annealed	0.42	0.17	0.55	0.25	0.35	Bal	-	-	-	-
5140	low-alloy structural steel	quenched and tempered	0.37	0.17	0.50	0.80	0.30	Bal	0.10	-	-	-
304	austenitic stainless steel	solution-annealed	0.08	1.00	2.00	19.0	10.2	Bal	-	-	-	-
420B	martensitic stainless steel	quenched and tempered	0.26	1.00	1.00	12.0	0.60	Bal	-	-	-	-
GH3625	nickel-based superalloy	Solution-treated	0.10	0.50	0.50	23.0	Bal	5.0	10.0	4.15	0.40	0.40

**Table 3:** The main physical properties of In625 powder and substrates [8].

	Coating	Substrate					
	In625	250S	1045	5140	304	GH3625	420B
Tensile strength, ***σ_b_***—MPa	760	250	600	980	515	830	735
Yield strength, ***σ_s_***—MPa	345	215	355	785	205	410	540
Young modulus, ***E***—GPa	205	130	209	211	194	205	219
Poisson ratio, **υ**	0.308	0.211	0.269	0.277	0.247	0.308	0.280
Melt point, ***T****—*°C	1350	1140	1480	1550	1450	1350	1500
Thermal expansion coefficient, **α**—10^−6^/°C	14.7	11.5	13.7	13.5	16.0	14.7	12.0

**Table 4:** The experimental results of pre-crack three-point-bending.

Order	Coating/Substrate	Ultimate Load, N	Critical Displacement, mm	Nominal Interface Strength, $\tilde{\tau_{C}}$ (MPa)
1	In625/250S	925	0.47	25.7
2	In625/1045	3570	1.18	99.2
3	In625/5140	3792	1.25	105.3
4	In625/304	4138	1.54	115.0

**Table 5:** The experimental results of rolling contact fatigue.

Order	Coating/Substrate	Cycle Times	Width, mm	Depth, μm
1	In625/1045	1.2 × 10^7^	0.96	17.3
2	In625/5140	1.2 × 10^7^	0.74	9.1
3	In625/304	1.2 × 10^7^	1.02	19.7
4	In625/GH3625	1.2 × 10^7^	0.83	14.9
5	In625/420B	4.4 × 10^6^	1.31	23.2

**Table 6:** Comparison between model and experimental results of In625/GH3625.

Coatings/Substrate	Model Results (MPa)	Experimental Results (MPa)	Relative Error (%)
In625/GH3625	115.9	110.4	5.0

**Table 7:** Evaluation parameters of interface critical damage model under different substrates.

Substrate	1045	5140	304	GH3625
***σ_yc_***, MPa	470	511	517	553
***τ_xyc_***, MPa	401	431	405	400
***C***	−0.0029	−0.0065	0.0082	−0.0199
***D_C_***	0.5715	0.5355	0.5768	0.5493
[**τ*_C_***], MPa	840	1266	730	1105

**Figure A1:**
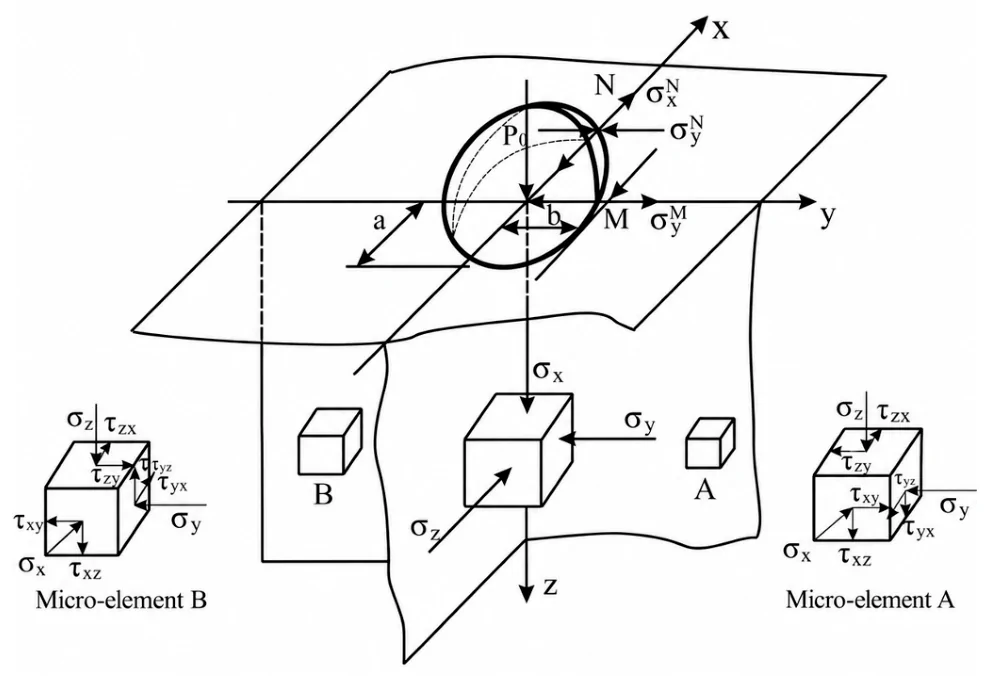
Schematic illustration of the stress state on the rolling contact surface and subsurface.

## Data Availability

The data presented in this study are available from the corresponding author upon reasonable request due to privacy and ethical restrictions.

## Appendix A

Although the Hertzian contact theory posits a frictionless surface assumption, it does not preclude its approximate application under frictional conditions. In rolling contact scenarios, the contact area of an elastic body typically assumes an elliptical shape, with stress distributed in accordance with an ellipsoidal pattern. The maximum contact stress is denoted as *P*_0_, and the configuration of contact stress is illustrated in Figure A1. The relationships among the stress components on the contact surface are delineated as follows: ① The normal stresses *σ_x_*, *σ_y_* and *σ_z_* manifest as compressive stresses, with the surface undergoing pulsating cyclic compressive stress during the rolling process. Points along the Z axis experience no shear stress, and the stress along this axis constitutes the principal stress. Away from the contact center, the values of *σ_x_*, *σ_y_* and *σ_z_* diminish to zero, while the stress value along the Z axis reaches its maximum. ② The signs of the orthogonal shear stresses *τ_xy_* and *τ_yx_* are contingent upon the sign of the product of the point’s positional coordinates X and Y. These stresses reduce to zero when located away from the contact center or when x = 0 or y = 0. Throughout the rolling process, these two stresses exhibit alternating characteristics. ③ The sign of the orthogonal shear stress *τ_zx_* is determined by the sign of the point’s positional coordinate X, becoming zero when situated away from the contact center or at X = 0. Similarly, the sign of *τ_yz_* is governed by the coordinate Y, also equating to zero when located away from the contact center or at Y = 0. Both of these shear stress components similarly demonstrate alternating stress behavior.

When two objects are loaded along the Z-axis direction, the comprehensive curvature radius R of the contact area subsequent to elastic deformation is:

(A1) $$\frac{1}{R}=\left(\frac{1}{R_{1x}}+\frac{1}{R_{1y}}\right)+\left(\frac{1}{R_{2x}}+\frac{1}{R_{2y}}\right)$$

The equivalent elastic modulus E*, the value of which can be calculated using the following formula:

(A2) $$\frac{1}{E^{*}}=\frac{1-\upsilon_{1}^{1}}{E_{1}}+\frac{1-\upsilon_{2}^{2}}{E_{2}}$$

When the rolling contact occurs between a spherical body and a planar surface, with *R*_2*x*_ and *R*_2*y*_ approaching infinity, the contact surface reduces to a circular form. In this case, the major and minor axes of the contact area are equal, denoted by ‘a’ as its radius, and *P*_0_ represents the maximum contact stress. Consequently, the following formula can be derived:

(A3) $$a={\left(\frac{3\mathrm{WR}}{4E^{*}}\right)}^{\frac{1}{3}},\;{\;P}_{0}=\frac{3W}{2\pi a^{2}}$$

In the equation, W signifies the external load, while R represents the radius of the rolling sphere. Given that the maximum shear stress *τ_max_* consistently lies on the line forming a 45° angle between the contact centerline and the principal stress direction, its magnitude is directly proportional to the maximum contact stress *P*_0_. Furthermore, the depth z at which the maximum shear stress occurs is also directly proportional to the contact radius R. The following formula is derived based on the maximum shear stress *τ_max_* and the corresponding maximum depth z.

*τ_max_* = 0.32*P*_0_, *z* = 0.47*a* (A4)
